# Single-cell epigenome analysis reveals age-associated decay of heterochromatin domains in excitatory neurons in the mouse brain

**DOI:** 10.1038/s41422-022-00719-6

**Published:** 2022-10-07

**Authors:** Yanxiao Zhang, Maria Luisa Amaral, Chenxu Zhu, Steven Francis Grieco, Xiaomeng Hou, Lin Lin, Justin Buchanan, Liqi Tong, Sebastian Preissl, Xiangmin Xu, Bing Ren

**Affiliations:** 1 Ludwig Institute for Cancer Research, La Jolla, CA USA; 2 School of Life Sciences, Westlake University, Hangzhou, China; 3 Bioinformatics and Systems Biology Graduate Program, University of California San Diego, La Jolla, CA USA; 4 Department of Anatomy and Neurobiology, School of Medicine, University of California, Irvine, CA USA; 5 Center for Epigenomics, University of California San Diego, La Jolla, CA USA; 6 Institute of Experimental and Clinical Pharmacology and Toxicology, Faculty of Medicine, University of Freiburg, Freiburg, Germany; 7 The Center for Neural Circuit Mapping, University of California, Irvine, CA USA; 8 Department of Cellular and Molecular Medicine, University of California San Diego School of Medicine, La Jolla, CA USA; 9 Institute for Genomic Medicine, University of California San Diego, La Jolla, CA USA

**Keywords:** Histone post-translational modifications, Gene silencing

## Abstract

Loss of heterochromatin has been implicated as a cause of pre-mature aging and age-associated decline in organ functions in mammals; however, the specific cell types and gene loci affected by this type of epigenetic change have remained unclear. To address this knowledge gap, we probed chromatin accessibility at single-cell resolution in the brains, hearts, skeletal muscles, and bone marrows from young, middle-aged, and old mice, and assessed age-associated changes at 353,126 candidate *cis*-regulatory elements (cCREs) across 32 major cell types. Unexpectedly, we detected increased chromatin accessibility within specific heterochromatin domains in old mouse excitatory neurons. The gain of chromatin accessibility at these genomic loci was accompanied by the cell-type-specific loss of heterochromatin and activation of LINE1 elements. Immunostaining further confirmed the loss of the heterochromatin mark H3K9me3 in the excitatory neurons but not in inhibitory neurons or glial cells. Our results reveal the cell-type-specific changes in chromatin landscapes in old mice and shed light on the scope of heterochromatin loss in mammalian aging.

## Introduction

Aging is a major risk factor for cardiovascular diseases, cancer, neurodegenerative diseases, type II diabetes and a variety of other common illnesses.^1^ Understanding the aging process at the molecular and cellular level is necessary for developing approaches to delay or prevent these late-onset age-related diseases. Recent research has uncovered molecular hallmarks of aging,^2^ including genomic instability,^3^ telomere attrition,^4^ loss of proteostasis,^5^ mitochondrial dysfunction,^6^ and epigenetic alterations.^7^ In particular, progressive changes to the epigenome, such as loss of histone proteins,^8,9^ altered patterns of histone modifications,^10^ DNA methylation,^11,12^ and expression of noncoding RNAs,^12,13^ occur in the process of aging. Most strikingly, the methylation levels of several hundred CpG sites across tissues can predict biological age in humans, dogs and mice.^14^ Furthermore, functional studies in model organisms have linked various epigenetic modifiers to the lifespan, specifically histone 3 lysine 4 tri-methylation (H3K4me3), H3K27me3 and histone acetylations.^8,15–17^ These studies raised the possibility that epigenetic mechanisms may contribute to aging in different organisms.

Chromosomes in eukaryotic cells are generally partitioned into transcriptionally active euchromatin and transcriptionally repressed heterochromatin compartments.^18^ The heterochromatin compartments are associated with hyper-methylation of lysine 9 on histone H3 (H3K9me2 and H3K9me3),^19^ and are generally located at the nuclear periphery and associated with the nuclear lamina.^20^ In one model of aging,^21^ erosion of heterochromatin domains is proposed to lead to de-repression of endogenous retrotransposons contained within those domains,^22,23^ leading to dysregulated immune responses^24–26^ and decline in organ functions. Supporting this model, global loss of heterochromatin has been observed during natural aging in *C. elegans*,^27^ *Drosophila*,^28^ mouse,^29^ and human.^30,31^ In addition, loss of heterochromatin has also been reported in pre-mature aging models^32–34^ and with cellular senescence.^22,35,36^ However, the cell types and genes subject to heterochromatin loss in the mammalian aging process remain to be characterized.

Phenotypically, the rates and extent of aging vary considerably depending on the cell types and tissue contexts. Therefore, molecular characterization at the bulk level inevitably fails to capture the heterogeneity of changes that aging inflicts in each cell type. Instead, single-cell approaches are required for molecular characterization of the distinct epigenomes in different cell types across the lifespan. Single-cell transcriptomic profiling studies have been performed on aging mammalian tissues,^37–41^ finding age-related changes in cellular composition, accumulation of senescent cells, increased transcriptional noise in aged cells, aging signatures common across many cell types, as well as features unique to each cell type.

To gain a deeper understanding of the transcriptional regulation during aging, it is necessary to also profile the epigenomes across the life span. In this study, we used the single-nucleus Assay for Transposase-Accessible Chromatin with sequencing (snATAC-seq) to study age-dependent changes of chromatin accessibility at single cell resolution in mouse frontal cortex, hippocampus, heart, bone marrow and skeletal muscles. Probing a total of 227,529 cells from these tissues in 3-month-, 10-month- and 18-month-old male mice, we determined alterations of the chromatin landscape in 32 major murine cell types during aging. We observed age-dependent changes in 77,881 candidate *cis*-regulatory elements (cCREs), most of which were found in the brain cell types. We therefore analyzed gene expression and the heterochromatin mark H3K9 tri-methylation (H3K9me3) in the mouse brain tissues using both single nucleus RNA-seq and Paired-Tag, a single cell multi-omics assay designed to profile both histone modification and nuclear transcriptome from the same cells. Unexpectedly, we discovered that many heterochromatin domains in the excitatory neurons in the old mice gain chromatin accessibility and lose H3K9 tri-methylation. This change is accompanied by increased transcription of noncoding RNA species as well as reduced nuclear staining of lamin B in these cells. Our results clarify genomic loci and cell types affected by heterochromatin loss in mammalian aging and suggest the potential effects of this epigenetic process on excitatory neurons.

## Results

### Single-nucleus ATAC-seq analysis of diverse tissues during aging in the mouse

**Fig. 1:**
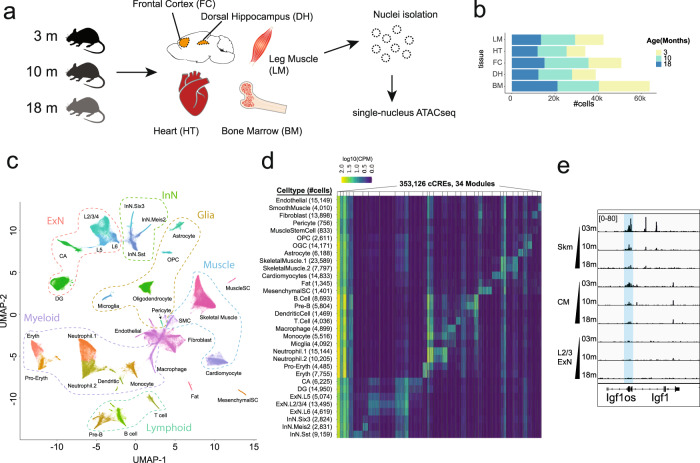
Overview of the snATAC-seq analysis of aging in the mouse. **a** Workflow of the tissue collection and snATAC-seq experiments. **b** Barplots showing the number of nuclei analyzed per tissue, colored by age groups. **c** Uniform Manifold Approximation and Projection (UMAP) plot of snATAC-seq data from all the nuclei profiled in the five tissues. **d** Heatmap showing the K-means (*K* = 34) clustering on the logarithm transformed (base 10) counts per million (CPM) signals of cCREs identified in the current study. **e** Genome browser view showing the chromatin accessibility at the *Igf1* locus for different cell types. Skm skeletal muscle, CM cardiomyocytes, L2/3 ExN layer-2/3 cortical excitatory neurons.

We collected the dorsal hippocampus, frontal cortex, heart, leg muscles and bone marrow from male C57BL/6JN mice in three age groups, namely 3-month, 10-month, and 18-month (Fig. 1a). These tissues were selected because they represent a diverse set of micro-environments with a mixture of post-mitotic (neurons, cardiac muscle cells) and mitotic (glia, fibroblast, and blood) cell types, that are associated with a broad spectrum of age-related diseases in humans. We performed single-cell combinatorial indexing (sci)-ATAC-seq^42^ with each tissue using two independent biological replicates. A total of 227,529 nuclei passed quality control measures, averaging ~15,000 nuclei per tissue-age and a median number of 8341 fragments per nucleus (Fig. 1b; Supplementary information, Fig. S1). We grouped them into 32 major cell types with the software package SnapATAC.^43^ The cell classes include excitatory neurons (ExN, 43,571 nuclei), inhibitory neurons (InN, 14,721 nuclei), glia (29,911 nuclei), muscle cells (43,897 nuclei), lymphoid cells (17,911 nuclei) and myeloid cells (50,286 nuclei), with the remaining 25,799 nuclei classified as “Other” (Fig. 1c). We annotated these cell types based on chromatin accessibility at the promoter and gene body of up to three marker genes per known cell type (Supplementary information, Fig. S2 and Table S1).

To systematically characterize the regulatory program of each cell type and to understand their age-related changes, we first determined the open chromatin regions of the candidate *cis*-regulatory elements (cCREs) in each of the 32 major cell types. We identified peaks using MACS2 software,^44^ generating a catalog of 353,126 open chromatin regions (7.0% of the mouse genome) from all cell types after merging overlapping peaks. The cCREs were highly enriched for active chromatin states or CTCF binding sites previously mapped by bulk analysis from ENCODE (Supplementary information, Fig. S2b). As expected, a majority of the cCREs displays cell-type-specific accessibility (Fig. 1d). While the chromatin landscape in most cell types were not drastically altered during aging (Supplementary information, Fig. S2c), mild to modest changes in chromatin accessibility at select gene loci in specific cell types are observed. For example, chromatin accessibility at the promoter and gene body of *Igf1* (insulin-like growth factor 1), a well-known regulator of the aging process,^45^ is significantly reduced in skeletal and cardiac myocytes during aging (Fig. 1e).

### Age- and tissue-associated changes of chromatin accessibility in different cell types

**Fig. 2:**
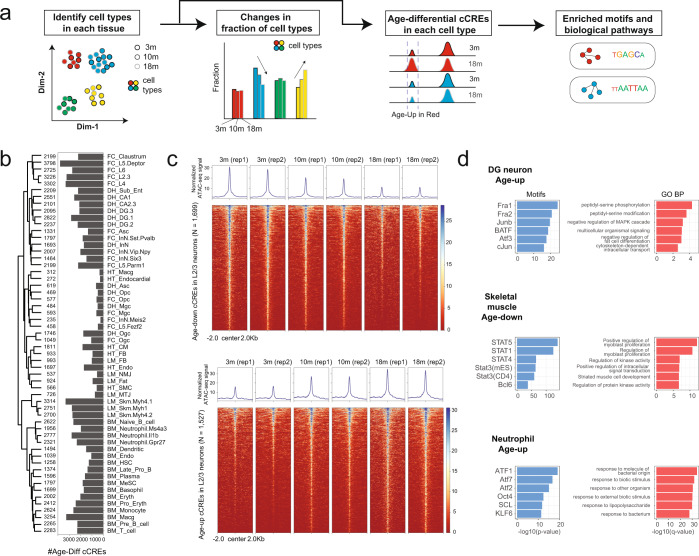
Cell-type specific changes in chromatin accessibility in different mouse tissues during aging. **a** Schematic diagram of the data analysis strategy to identify changes in cell type composition, age-dependent cCREs, enriched transcription factor motifs and biological pathways for each cell type. See Materials and Methods for details. **b** A bar-plot showing the number of age-dependent cCREs in a few major cell types. The dendrogram was calculated based on the pairwise Jaccard index between age-dependent cCREs from two cell types. **c** Heatmaps showing the chromatin accessibility signal in age-downregulated (top) and age-upregulated (bottom) cCREs in layer-2/3 cortical neurons. **d** Bar-plots showing transcription factor motifs and GO biological pathways enriched in age-up or age-down cCREs in three representative cell types.

We next performed tissue-level clustering (Supplementary information, Figs. S3, S4 and Table S1) and queried if there was any change in the relative fraction of cell types (Fig. 2a). Although some changes in cell type fractions are observed (Supplementary information, Fig. S5), such as an increased fraction of Naïve B cells and T cells in aging bone marrow, more biological replicates are needed to ascertain the statistical significance of such changes. On the other hand, for each cell type, we identified differentially accessible cCREs between different age groups (Fig. 2b; Supplementary information, Fig. S4d). To ensure the robustness of the results, we tested three computational approaches: 1) comparing 3-month and 18-month samples with edgeR;^46^ 2) comparing all three age groups with edgeR; and 3) using a linear regression model MAST^47^ and treating age as a numerical variable. All of these approaches showed comparable results (Supplementary information, Fig. S6), except that edgeR comparing all age groups identified some cCREs unique to 10-month mice (Supplementary information, Fig. S7). Here we report the edgeR differential testing results between 3-month and 18-month samples as it generated the most consistent results (see Materials and Methods). A total of 77,881 cCREs were found to be differentially accessible between 3-month-old and 18-month-old mice in at least one cell type (Fig. 2b). Of these, 39,285 and 26,382 cCREs show age-dependent accessibility in the brain and bone marrow, respectively, whereas only 7,216 and 11,745 cCREs show changes in chromatin accessibility in heart and leg muscle, respectively. These cCREs generally display continuous and reproducible gain or loss of accessibility during aging (Fig. 2c). Because the power to detect differential cCREs depend on the number of cells and sequencing depth of each cell type, to ensure a fair comparison of age-associated changes among different cell types, we down-sampled snATAC-seq data of each cell type in each sample to 1 million reads (cell types with less than 1 million reads per sample were removed and 32 out of 75 subtypes were analyzed after down-sampling) and performed differential testing (Supplementary information, Fig. S8). Cell types in the heart (cardiomyocytes and endothelial cells) display the smallest number of age-dependent cCREs, and there was no significant difference in the number of age-dependent cCREs between mitotic and post-mitotic cells (Supplementary information, Fig. S8).

Overall, 73% of the age-dependent changes in chromatin accessibility at cCREs was cell-type specific, but closely related cell types tend to share common age-dependent changes (Supplementary information, Fig. S9). Motif and gene set enrichment analysis on these dynamic cCREs revealed putative transcription factors (TF) and biological pathways that may be dysregulated during aging in these cell types (Fig. 2a; Supplementary information, Tables. S2, S3). For instance, DNA recognition motifs of the Jun/Fos/AP-1 family of TFs were enriched in the cCREs with increased chromatin accessibility in many neuronal cell lineages in the old mice (Supplementary information, Table S2) such as the dentate gyrus (DG) neurons (Fig. 2d), and the genes near these cCREs are enriched for those involved in peptidyl-serine modification (Fig. 2d). Interestingly, chronic AP-1 activation during aging was recently shown to promote human tau pathology and degeneration.^48^ On the other hand, DNA binding motifs of downstream effectors of the JAK/STAT pathway^49^ are highly enriched in cCREs showing reduced accessibility in skeletal muscle cell types in the old mice (Fig. 2d), and genes near these cCREs were enriched for those involved in myoblast proliferation (Fig. 2d). In neutrophils, DNA binding motifs of the ATF family of TFs were enriched in cCREs with increased chromatin accessibility during aging (Fig. 2d), and the genes near these cCREs were enriched for immune response related pathways. The ATF family of transcription factors respond to extracellular signals and maintain homeostasis, and several of its members, such as ATF3 and ATF7, have been implicated in immune responses.^50,51^

**Fig. 3:**
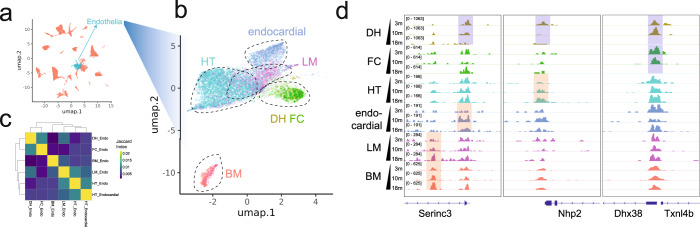
Age-dependent changes of chromatin accessibility at cCREs from endothelial cells. **a** UMAP plot showing cells from all tissues with endothelial cells highlighted. **b** UMAP plot showing the sub-clustering results of endothelial cells, colored by tissue origin. Endocardial cell is a special type of endothelial cell present in heart. **c** Heatmap showing the pairwise Jaccard Index between age-differential cCREs from different endothelial cell subtypes. **d** Genome browser view showing the change in chromatin accessibility at a few loci for all subtypes of endothelial cells. Blue shades mark age-down cCREs and red shades mark age-up cCREs.

We also observed that age-associated chromatin changes in some cell types can be tissue-dependent. For example, the overlap of age-dependent cCREs between endothelial cells from different tissues is very low (< 1% overlap between cell types; Fig. 3a–c), suggesting that a substantial number of age-dependent changes of chromatin state at cCREs might be driven by the local microenvironment (Fig. 3d). For instance, the promoter of *Nhp2* is more accessible in the endothelial cells in the heart of the old mice, whereas no change is observed in other tissues, except for a slight decrease in dorsal hippocampus (Fig. 3d). *Nhp2* encodes a protein subunit for the H/ACA ribonucleoprotein complex required for ribosome biogenesis and telomere maintenance. Mutations in this gene can cause the premature aging syndrome dyskeratosis congenita.^52^ Taken together, our data identify cell-type-specific and tissue environment dependent changes in chromatin landscape during mouse aging.

### Transcriptional changes in cortical and hippocampal cell types during aging

**Fig. 4:**
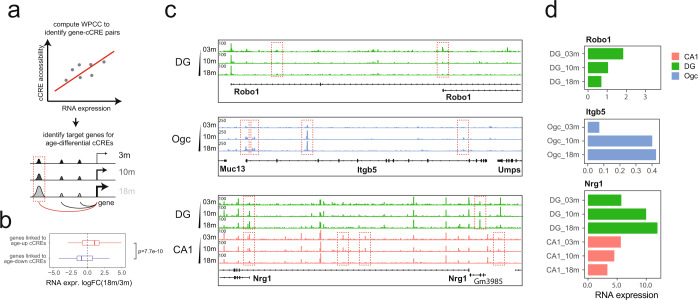
Predicted target genes for age-dependent cCREs. **a** A schematic overview of the computational strategies to identify cCREs whose activity positively correlated with transcription of target genes, and to link age-differential cCREs to age-differential genes. WPCC, Weighted Pearson Correlation Coefficient. **b** Box-plot showing the RNA expression fold change (18-month over 3-month) of the genes which are linked to age-up or age-down cCREs. **c** Genome browser view of the chromatin accessibility signal in *Robo1*, *Itgb5*, and *Nrg1* loci. Red dashed boxes indicate age-differential cCREs linked to *Robo1*, *Itgb5* and *Nrg1*. **d** Average single-cell gene expression of *Robo1*, *Itgb5* and *Nrg1* for each indicated cell type and age.

Because much of the age-dependent changes in chromatin state among the tissues examined in the current study occurred in the dorsal hippocampus and the frontal cortex, which play central roles in behavioral and cognitive functions, we focused the subsequent analyses on these two brain regions. We first examined whether the age-associated changes in chromatin accessibility are accompanied by transcriptional alterations. We obtained snRNA-seq data from all three age groups (Supplementary information, Fig. S10). We first identified clusters with snRNA-seq data, and performed joint clustering with the snATAC-seq data from frontal cortex and hippocampus from the same age groups, using Seurat’s anchor-based method^53^ (Supplementary information, Fig. S10b, f). For each joint cluster, we aggregated the ATAC-seq and RNA-seq signals, and calculated a weighted Pearson correlation coefficient (WPCC) between the cCRE accessibility (count per million DNA fragments) and transcription levels of gene (count per million transcripts) within 500kbp of the cCRE (Fig. 4a). cCRE-gene pairs reaching a significant correlation (adjusted *P*-value less than 0.05) were used to predict potential target genes for age-differential cCREs in each cell type (Fig. 4a). As expected, predicted target genes of cCREs that gained chromatin accessibility in old animals tended to show increased transcription levels during aging, whereas predicted target genes of cCREs losing chromatin accessibility tended to show decreased transcription (Wilcoxon rank sum test, *P*-value = 7.7E-10) (Fig. 4b). Altogether, we identified 474 cCRE-gene pairs showing cell-type-specific and concordant changes in accessibility and gene expression during aging. For instance, in DG neurons, we observed reduced accessibility in a putative enhancer and an alternative promoter which could explain the decrease of Robo1’s activity during aging (Fig. 4c, d, upper row). Robo1 plays an important role in midline axonal guidance.^54^ In oligodendrocytes, we observed increased accessibility at putative enhancers and the promoter of *Itgb5*, accompanied by an increase of *Itgb5* RNA expression during aging (Fig. 4c, d, mid row). *Itgb5* encodes a beta subunit of integrin, which mediates cell-to-cell and cell-to-extracellular matrix interactions. Integrins have been implicated in cellular senescence and aging.^55,56^ More interestingly, at the *Nrg1* gene locus (an important neurotrophic growth factor and potentially associated with rodent longevity^57^), we identified cCREs that display increased accessibility in DG neurons and a different set of cCREs that lose accessibility in CA1 neurons during aging, which may contribute to its opposite expression change in corresponding cell types (Fig. 4c, d, bottom row). Bi-directional aging signatures that show opposite directions of change in different cell types have been described in a single-cell brain transcriptomic study.^39^ Here, by dissecting the cell-type-resolved DNA accessibility maps, we further revealed the molecular mechanisms that underlie transcriptional alterations, including such bi-directional changes.

### Increased chromatin accessibility in selective heterochromatin domains in excitatory neurons during aging

**Fig. 5:**
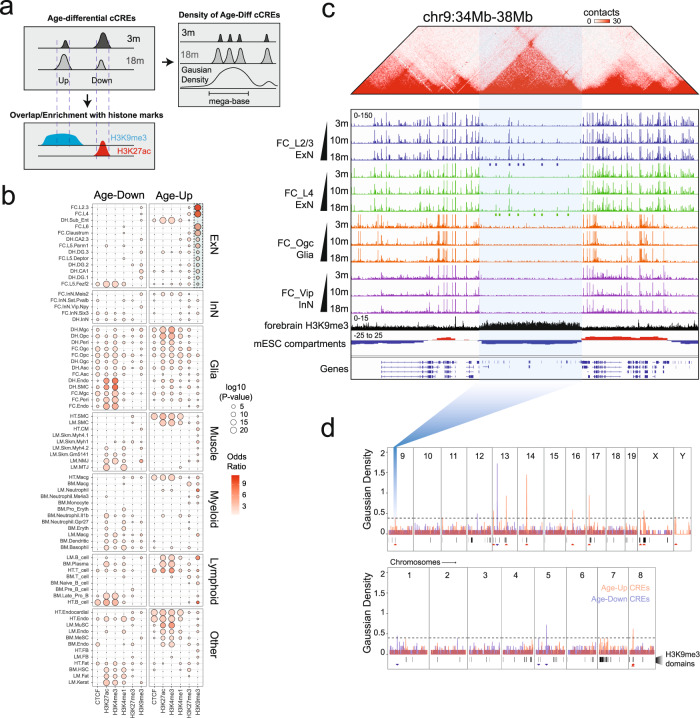
Age-up cCREs in excitatory neurons are enriched in heterochromatin domains. **a** Schematic workflow showing how to detect overlap and enrichment of age-differential cCREs with histone marks and how to detect large clusters of age-differential cCREs. **b** Bubble plot showing the enrichment of the overlap between age-up or age-down cCREs with each type of histone modifications and CTCF. **c** Genome browser view of the ATAC-seq signals of a few cell types in the brain, H3K9me3 signals from post-natal forebrain and compartment (first principal component from Hi-C) and Hi-C matrix from mouse embryonic stem cells.^94^ Blue and green rectangles indicate locations of age-up cCREs in corresponding cell types. **d** Genomic view of cell-type-specific gaussian density score of age-up or age-down cCREs from all brain cell types, along with H3K9me3 domains from post-natal forebrain. Red and blue triangles indicate age-up or age-down cCRE cluster, respectively.

By comparing the age-dependent cCREs to the previous profiles of histone modifications and CTCF binding of the same mouse tissues^58^ (Fig. 5a), we found enrichment of cCREs gaining chromatin accessibility in excitatory neurons in old animals in the constitutive heterochromatin domains (H3K9me3) identified in fetal and perinatal mouse brains (Fig. 5b). These cCREs appear in clusters that overlap with inactive chromatin compartments which are demarcated by topologically associating domain boundaries^59–61^ (Fig. 5c; Supplementary information, Fig. S11). To comprehensively characterize such cCRE clusters genome-wide, we calculated a Gaussian density score of the age-up (accessibility increases with age) and age-down (accessibility decreases with age) cCREs in each cell type in the dorsal hippocampus and frontal cortex (Fig. 5a). We identified 15 cCRE clusters (mean size of 1.22 M base pairs) that show age-dependent chromatin accessibility change, with eleven gaining accessibility and four losing accessibility (Fig. 5d). Ten of the eleven age-up cCRE clusters were found in excitatory neurons, and nine of them overlap with H3K9me3 domains (Fig. 5d). While 3% of the genome is covered by H3K9me3 domains in mouse forebrain, about 12% of these domains overlap with age-up cCRE clusters, a percentage that is more than 21-fold higher than would be expected by chance (0.6%).

### Reduced H3K9me3 heterochromatin in excitatory neurons during mouse aging

**Fig. 6:**
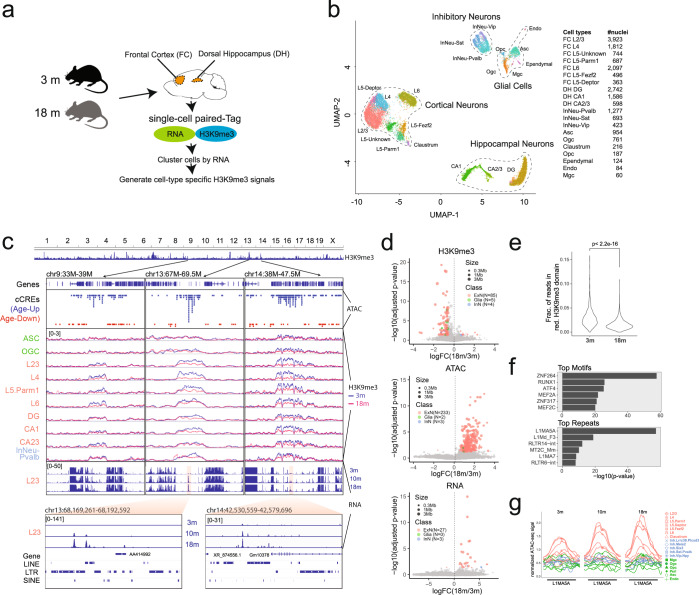
Loss of heterochromatin domains in excitatory neurons. **a** Schematic diagram of the tissue collection, Paired-tag assays, and data analysis strategies. **b** UMAP plot of the cells based on RNA component of the Paired-tag data. **c** Genome browser view showing the H3K9me3 signal, age-dependent cCREs (from dorsal hippocampus and frontal cortex), and snRNA-seq signal (of layer-3 cortical neurons) at three different loci for a few representative cell types. Close-up views of the snRNA-seq signal in two loci were shown at the bottom. **d** Volcano scatter plot showing the negative logarithmic transformed adjusted *P*-value and logarithmic transformed fold change of H3K9me3, ATAC-seq and RNA-seq signals between 18-month and 3-month for each H3K9me3 domain and each cell type. Each circle is one domain in one cell type. **e** Violin plot showing the distribution of the fraction of reads in age-reduced H3K9me3 domain in 3-month and 18-month layer-2/3 cortical neurons. **f** Bar-plots showing the enrichment of transcription factor motifs and repetitive elements in the age-up cCREs that overlap with age-reduced H3K9me3 domains. **g** Aggregated ATAC-seq signal over L1MA5A elements in all cell types from frontal cortex.

The gain of chromatin accessibility in heterochromatin domains in the excitatory neurons raised the possibility that heterochromatin may be lost in these cells during aging. To directly test this hypothesis, we performed Paired-tag^62^ experiments with the frontal cortex and dorsal hippocampus from 3-month- and 18-month-old mice, to profile jointly gene expression and H3K9me3 at single-cell resolution in the two brain regions. After filtering, we obtained joint profiles of H3K9me3 (median: 2,517 unique fragments per nucleus) and RNA profiles (median: 1,785 unique molecular identifier per nucleus, median 323 genes) from 19,827 nuclei (Fig. 6a). We clustered the cells based on their transcriptomes and assigned cell type labels based on the marker genes as previously defined^62^ (Fig. 6a, b). Then we aggregated signals from cells of the same cluster to assess the genomic distribution of H3K9me3 in each cell type and age group (Fig. 6a, c). Supporting the above hypothesis, we observed reduced H3K9me3 levels in excitatory neurons within heterochromatin domains overlapping cCRE clusters that gained accessibility during aging (Fig. 6c). We further found elevated transcription levels of RNA species in these regions in the excitatory neurons. The derepressed RNA species were primarily unannotated, and appeared to have derived from transcripts made from the repetitive elements (Fig. 6c), or pseudogenes (e.g. *Gm16505* in Supplementary information, Fig. S12a).

Furthermore, genome-wide analysis of all 642 H3K9me3 domains revealed reduced H3K9me3 levels, gain of DNA accessibility, and increased transcription during aging in a subset of the domains. Strikingly, these changes predominantly occurred in excitatory neurons (Fig. 6d). We compared the domains with reduced H3K9me3 to domains with no change during aging. Interestingly, the domains with reduced H3K9me3 signals show a higher level of H3K9me3 signals than the stable domains in the young mice. However, in 18-month-old mice, this difference between these two types of heterochromatin domains becomes negligible (Supplementary information, Fig. S13a). Some transposable elements (TEs) and gene sets are enriched in the domains losing H3K9me3 in old mice, such as “antibacterial humoral response” that comprise of defensin beta gene cluster and “cell adhesin” which contains the protocadherin gene cluster (Supplementary information, Fig. S13b, c and Table S4). To determine if the reduction of H3K9me3 is occurring in all cells or in a subset of cells as they go through an age-related process such as cellular senescence, we calculated the fraction of reads within the reduced H3K9me3 domains during aging in layer-2/3 cortical neurons. We observed a uniform decrease of H3K9me3 signals in the 18-month mice, suggesting that H3K9me3 at these domains is likely reduced across all layer-2/3 excitatory neurons at the same pace (Fig. 6e). We also examined the expression of 60 marker genes associated with senescence or senescence-associated secretory phenotype (Supplementary information, Table. S5), but did not observe significant up-regulation of senescence marker genes in aged excitatory neurons (Supplementary information, Fig. S14).

We further investigated the sequence features of the cCREs gaining accessibility that reside within the H3K9me3 domains. It revealed a significant enrichment of sub-families of LINE-1 and ERV elements (Fig. 6f). In line with this, enrichment of two Krüppel-associated box domain-containing zinc finger protein (KZFP) motifs was observed in these age-up cCREs. KZFPs are the largest family of transcription factors in tetrapod vertebrates, a majority of whose function is to silence retrotransposons.^63^ Interestingly, the most enriched LINE-1 element, L1MA5A, is already accessible in the excitatory neurons of the 3-month-old mice, and the accessibility further increases during aging (Fig. 6g). This observation is consistent with the previous reports that LINE-1 elements are active in neurons,^64,65^ and transposable elements are re-activated during aging.^66,67^ Analysis of the snRNA-seq data confirmed that repetitive elements are more active in neurons than in glial cells (Supplementary information, Fig. S15). However, we did not observe significant transcriptional up-regulation in L1MA5A or other LINE1 elements in 18-month-old mice (Supplementary information, Fig. S16a, b). This might be due to the sensitivity of detection by current snRNA-seq techniques. Transcription and chromatin accessibility of some LTR elements are up-regulated in aged mice, however, they are not accompanied by reduction of H3K9me3-associated heterochromatin (Supplementary information, Fig. S16).

**Fig. 7:**
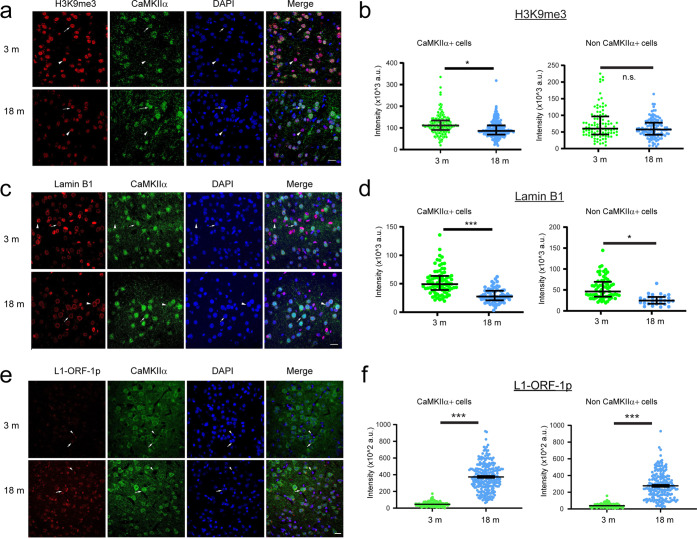
Age-dependent changes in H3K9me3, Lamin B1 and LINE-1-ORF-1p immunostaining in cortical excitatory neurons. **a** Images showing H3K9me3 immunoreactivity in layer-2/3 of frontal cortex of 3-month and 18-month-old mice. Scale bar, 20 μm. The cells immunopositive for both H3K9me3 and CaMKIIα, or H3K9me3 only are indicated by the arrow or arrowhead respectively. **b** Left: The violin plot graph and quantification of H3K9me3 staining intensity in CaMKIIα positive cells in the frontal cortex of 3-month- and 18-month-old mice. The median, and 25^th^ and 75^th^ percentile values are plotted in the violin graph. *P = 0.014. Right: The violin plot graph and quantification of H3K9me3 staining intensity in non-CaMKIIα cells in the frontal cortex of 3-month- and 18-month-old mice. n.s, *P* = 0.075. **c** Images showing Lamin B1 immunoreactivity in layer-2/3 of frontal cortex of 3-month- and 18-month-old mice. The cells immunopositive for both Lamin B1 and CaMKIIα or Lamin B1 only are indicated by the arrow or arrowhead respectively. Scale bar, 20 μm. **d** Left: The violin plot graph and quantification of Lamin B1 staining intensity in CaMKIIα positive cells in the frontal cortex of 3-month- and 18-month-old mice. ***P = 0.00029. Right: The violin plot graph and quantification of Lamin B1 staining intensity in non-CaMKIIα cells in the frontal cortex of 3-month- and 18-month-old mice. *P = 0.016. **e** Images showing LINE-1-ORF-1p immunoreactivity in layer-2/3 of frontal cortex of 3-month- and 18-month-old mice. Scale bar, 20 μm. The cells immunopositive for both LINE-1-ORF-1p and CaMKIIα or LINE-1-ORF-1p only are indicated by the arrow or arrowhead respectively. **f** Left: The violin plot graph and quantification of LINE-1-ORF-1p staining intensity in CaMKIIα positive cells in the frontal cortex of 3-month and 18-month-old mice. Right: The violin plot graph and quantification of LINE-1-ORF-1p staining intensity in non CaMKIIα cells in the frontal cortex of 3-month- and 18-month-old mice. ***P < 0.00001.

To confirm the loss of heterochromatin in cortical excitatory neuron during aging, we performed immunostaining of H3K9me3 in the frontal cortex sections of young (3-month-old) and aged (18-month-old) mice. We utilized the antibody against CaMKIIα to label the excitatory neurons and DAPI staining to locate the nuclei. Consistent with the results from the Paired-Tag experiments, we observed a significant decrease of H3K9me3 immunoreactivity in excitatory neurons in cortical layer-2/3 of frontal cortex of 18-month-old mice compared to young mice (immunofluorescent intensity median: 111 × 10^3^ a.u. vs 86 × 10^3^ a.u., linear mixed-effect model (LME) using “age” for a fixed effect and “mouse group” for a random effect, *P* = 0.014, Fig. 7a, b). We also observed an age-dependent change in the nuclear staining pattern of H3K9me3 in excitatory neurons, evidenced by increased aggregates of H3K9me3 staining within the nuclei of excitatory neurons in aged mice (Fig. 7a). By contrast, no significant difference in H3K9me3 immunoreactivity was observed in non-CaMKIIα cells (immunofluorescent intensity median: 60 × 10^3^ a.u. vs 57 × 10^3^ a.u., *P* = 0.075, LME, Fig. 7b). This result is consistent with our observation of H3K9me3 domain decay specifically in excitatory neurons.

We next examined the level of Lamin B1 in young and aged mice. There was a robust decrease in immunoreactivity of Lamin B1 in excitatory neurons in frontal cortex of aged mice (immunofluorescent intensity median: 49 × 10^3^ a.u. for cells in young mice vs 27 × 10^3^ a.u. for cells in aged mice, *P* = 0.00029, LME, Fig. 7c, d). The same trend for Lamin B1 can be observed in non-excitatory cells (immunofluorescent intensity median: 46 × 10^3^ a.u. for cells in young mice vs 24 × 10^3^ a.u. for cells in aged mice, *P* = 0.016, LME, Fig. 7d). However, the age-dependent influence on H3K9me3 and Lamin B1 appears more pronounced in excitatory neurons. This decrease is not likely due to decreased transcription levels of Lamin B1 or other known regulators of H3K9me3, including *Setdb1*, *Ehmt2* and *Suv39h1/2*, as they were not significantly altered during aging (Supplementary information, Fig. S17).

We also examined the expression of the LINE-1 encoded protein, open reading frame 1 protein (LINE-1-ORF-1p) (Fig. 7e, f), in the frontal cortex of young (3-month-old) and aged (18-month-old) mice. We observed a significant increase of LINE-1-ORF-1p immunoreactivity in both CaMKIIα excitatory neurons and non-CaMKIIα cells in L2/3 of frontal cortex of aged mice compared with young mice (immunofluorescent intensity meridian: 40 × 10^2^ a.u. for CaMKIIα cells in young mice vs 364 × 10^2^ a.u. in aged mice, *P* = 9.3 × 10^−71^, LME; 34 × 10^2^ a.u. for non-CaMKIIα cells in young mice vs 250 × 10^2^ a.u. in aged mice, *P* = 2.5 × 10^−28^, LME, Fig. 7f). In addition, CaMKIIα^+^ cells have significantly higher level of LINE-1-ORF-1p than non-CaMKIIα cells in either group (immunofluorescent intensity meridian: 40 × 10^2^ a.u. for CaMKIIα^+^ cells vs 34 × 10^2^ a.u. for non-CaMKIIα cells in young mice, *P* = 0.03; 364 × 10^2^ a.u. for CaMKIIα^+^ cells vs 34 × 10^2^ a.u. for non-CaMKIIα cells in aged mice, *P* = 3.2 × 10^−6^, LME, Fig. 7f).

## Discussion

In this study, we investigated chromatin accessibility changes in brain, heart, skeletal muscle and bone marrow in the mouse at single-cell resolution. First, we showed that the cellular identity and composition did not change radically in the tissues that we analyzed, consistent with a previous report.^39^ We identified 77,881 age-dependent cCREs, and showed that the majority of them display age-dependent changes in chromatin accessibility in a cell-type restricted manner. By comparing age-dependent cCREs in different cell types and the same cell type in different tissues, we found that original epigenomic states of cells and tissue environment may both contribute to their differential epigenetic alterations during aging.

Through integrative analysis with published epigenetic maps from the same tissues, we revealed a decay of heterochromatin domains during aging in excitatory neurons, which we validated using single-cell multi-omics assays and immunostaining experiments. Previous microscopic studies revealed a global loss of H3K9me3-associated heterochromatin in aged animals or primary cells.^27–31^ However, a comprehensive survey of cell types and genetic elements affected by the loss of heterochromatin was lacking. Here we report that the loss of heterochromatin is restricted to selective neuronal cell types and genomic regions, at least for the time frame (from 3 months to 18 months) that we examined. The loss of heterochromatin in excitatory neurons, but not inhibitory neurons implies that a specific epigenetic program or neuronal activity may render excitatory neurons more susceptible to heterochromatin loss. As a result of the heterochromatin loss, these regions become de-repressed evidenced by an increase in chromatin accessibility. As heterochromatin regions are enriched for retrotransposons such as L1 elements, not surprisingly we found that some L1 elements become more accessible during aging in excitatory neurons. Although transcriptional changes were not observed at L1s in 18-month-old mice in this study, we observed higher protein level of LINE-1-ORF-1p in aged excitatory neurons and other cells in the mouse frontal cortex, consistent with previous reports in the human brain^68^ and senescent cells, mouse liver and muscle.^66,67^ Age-dependent up-regulation of L1 elements may promote age-associated inflammation,^66^ and potentially neuro-degeneration. Previous studies have suggested potential association of loss of heterochromatin and neurodegenerative disease.^69,70^ Future studies are needed to examine the relationship between the heterochromatin loss in the excitatory neurons and cognitive declines.

Apart from the excitatory neurons, we were unable to detect enrichment of age-dependent increase of chromatin accessibility at heterochromatin regions in cell types from other tissues. This could be due to our filtering strategy in the data processing step, where we remove cCREs that overlap with high-signal repetitive regions. This could filter out centromeric heterochromatin regions that may undergo age-dependent changes. The second reason could be that in our study, the oldest age was 18 months, which may be characterized as late middle age. Changes in the peripheral tissues such as bone marrow and muscle may become detectable in older mice.

In summary, our study complements recent single-cell transcriptomics studies by providing a resource for the study of the epigenome at cell type resolution during aging in the mouse. We revealed epigenetic alterations and heterochromatin loss, and integrated these epigenome atlases with transcriptomic data to understand the regulatory mechanisms responsible for the transcriptional changes during aging.

## Materials and Methods

### Mouse tissue dissection

Adult C57BL/6 J male mice were purchased from Jackson Laboratories (strain #000664). Tissues were extracted from 3-month-old, 10-month-old and 18-month-old mice. All dissections were performed consistently in sterile conditions by the same laboratory member. Briefly, prefrontal cortex and dorsal hippocampus were dissected in ice-cold ACSF (in mM: 126 NaCl, 2.5 KCl, 26 NaHCO_3_, 2 CaCl_2_, 2 MgCl_2_, 1.25 NaH_2_PO_4_, and 10 glucose). Both the prefrontal cortex and dorsal hippocampus were dissected out from both hemispheres of each mouse, using a brain block and scalpel as described before.^71,72^ Brain tissues were then immediately “flash frozen” in liquid nitrogen for down-stream applications. In similar fashion the heart and femoral bone and attached musculature were dissected from the animal. The entire heart was dissected, and flash frozen in liquid nitrogen. The quadriceps femoris muscle was dissected from the femur and flash frozen in liquid nitrogen. The femur was then further processed for obtaining bone marrow. Briefly, all tissue was removed from the femur. The distal end was then cut and placed in an eppendorf tube to be centrifuged at 4 °C. The bone marrow was then flash frozen in liquid nitrogen (Please see https://www.jove.com/t/53936/murine-hind-limb-long-bone-dissection-and-bone-marrow-isolation).

### Tissue preparation and nuclei isolation for snATAC-seq and snRNA-seq

#### Frontal cortex and dorsal hippocampus

For snATAC-seq on dorsal hippocampus and frontal cortex, tissue was homogenized using mortar and pestle on liquid nitrogen.^42^ ~20 mg ground tissue was suspended in 1 mL of nuclear permeabilization buffer: 5% BSA, 0.2% IGEPAL CA-630 (Sigma-Aldrich), 1 mM DTT, and 1× EDTA-free protease inhibitor (Roche or Pierce) in PBS). Nuclei were rotated at 4 °C for 5 min before being pelleted again with a swinging-bucket centrifuge (500× *g*, 5 min, 4 °C; 5920 R, Eppendorf).

For snRNA-seq on hippocampus, ~20 mg snap-frozen and ground hippocampus was suspended in 500 µL of nuclei buffer: 0.1% Triton-X-100 (Sigma-Aldrich, T8787), 1× EDTA free protease inhibitor (Roche or Pierce), 1 mM DTT, and 0.2 U/µL RNase inhibitor (Promega, N211B), 2% BSA (Sigma-Aldrich, SRE0036) in PBS. Sample was incubated on a rotator for 5 min at 4 °C and then pelleted with a swinging bucket centrifuge (500× *g*, 5 min, 4 °C; 5920 R, Eppendorf). For biological replicates of hippocampus, snATAC-seq and snRNA-seq were performed on aliquots of the same ground tissue. For the other biological replicate, starting tissue from different mice was used for snATAC-seq and snRNA-seq respectively.

For snRNA-seq on frontal cortex, ~20 mg snap-frozen and ground frontal cortex (Biological Replicate 2, aliquot from the same powder used for snATAC-seq) or a whole single snap-frozen frontal cortex were homogenized as described before with modifications.^73^ Tissue was transferred to a glass dounce and submerged in 1 mL dounce buffer: 0.25 M Sucrose (Sigma), 25 mM KCl, 5 mM MgCl_2_, Tris-HCl, pH 7.5, 1× EDTA-free protease inhibitor (Roche or Pierce), 1 mM DTT, and 0.2 U/µL RNase inhibitor (Promega, N211B), 2% BSA (Sigma-Aldrich, SRE0036) in PBS. Samples were homogenized using a loose pestle for 5–10 strokes followed by a tight pestle for 15–20 strokes. Suspension was transferred to a pre-chilled 1.5 mL LoBind tube (Eppendorf) through a 30 μM CellTrics filter (Sysmex) and pelleted with a swinging bucket centrifuge (100× *g*, 10 min, 4 °C; 5920 R, Eppendorf).

#### Heart and leg muscle tissue

Nuclei were isolated from individual snap-frozen whole heart and leg muscle tissue as described^74^ using gentleMACS M tubes (Miltenyi) on a gentleMACS Octo dissociator (Miltenyi). Tissue was submerged in magnetic-activated cell sorting (MACS) buffer: 5 mM CaCl_2_, 2 mM EDTA, 1× protease inhibitor EDTA-free (Roche or Pierce), 300 mM MgAc, 10 mM tris-HCl (pH 8.0), and 0.6 mM DTT (Sigma-Aldrich) and tissue was homogenized using the “Protein_01_01” protocol. Nuclei were pelleted with a swinging-bucket centrifuge (500 rcf, 5 min, 4 °C; 5920 R, Eppendorf) and resuspended in 1 mL of nuclei permeabilization buffer: 5% BSA, 0.2% IGEPAL CA-630 (Sigma-Aldrich), 1 mM DTT, and 1× EDTA-free protease inhibitor (Roche or Pierce) in PBS. Nuclei were rotated at 4 °C for 5 min before being pelleted again with a swinging-bucket centrifuge (500× *g*, 5 min, 4 °C; 5920 R, Eppendorf).

#### Bone marrow

Nuclei were isolated from individual snap-frozen bone marrow. 500 μL chilled OMNI buffer:^75^ 10 mM Tris-HCl, pH 7.5, 10 mM NaCl, 3 mM MgCl_2_, 0.1% IGEPAL CA-630 (Sigma-Aldrich), 0.1% Tween-20, 0.01% Digitonin (Promega) was added to the sample tube and a homogeneous suspension was obtained by gentle pipetting on ice. Suspension was transferred to a pre-chilled 1.5 mL LoBind tube (Eppendorf) through a 30 μM CellTrics filter (Sysmex). Sample tube was rinsed with another 500 μL chilled OMNI buffer and the suspension was transferred to the same LoBind tube through filter. The sample was kept on ice for 5 min and then pelleted with a swinging bucket centrifuge (500 rcf, 5 min, 4 °C; 5920 R, Eppendorf).

### snATAC-seq experiments

Combinatorial barcoding snATAC-seq was performed as described previously^43,74,76,77^ and the protocol for library preparation can be found here: https://www.protocols.io/edit/snatac-seq-library-generation-using-combinatorial-bpwcmpaw.

Pelleted and permeabilized nuclei were resuspended in 500 μL high salt tagmentation buffer (36.3 mM Tris-acetate, pH = 7.8), 72.6 mM potassium-acetate, 11 mM Mg-acetate, 17.6% DMF) and counted using a hemocytometer. Concentration was adjusted to 2000 nuclei/9 μL, and 2000 nuclei were dispensed into each well of one 96-well plate. For tagmentation, 1 μL barcoded Tn5 transposomes^43^ was added using a BenchSmart™ 96 (Mettler Toledo), mixed five times and incubated for 60 min at 37 °C with shaking (500 rpm). To inhibit the Tn5 reaction, 10 µL of 40 mM EDTA were added to each well with a BenchSmart™ 96 (Mettler Toledo) and the plate was incubated at 37 °C for 15 min with shaking (500 rpm). Next, 20 µL 2× sorting buffer (2% BSA, 2 mM EDTA in PBS) was added using a BenchSmart™ 96 (Mettler Toledo). All wells were combined into a FACS tube and stained with 3 µM Draq7 (Cell Signaling). Using a SH800 (Sony), 20 2n nuclei were sorted per well into eight 96-well plates (total of 768 wells) containing 10.5 µL EB: 25 pmol primer i7, 25 pmol primer i5, 200 ng BSA (Sigma). Preparation of sort-plates and all downstream pipetting steps were performed on a Biomek i7 Automated Workstation (Beckman Coulter). After addition of 1 µL 0.2% SDS, samples were incubated at 55 °C for 7 min with shaking (500 rpm). 1 µL 12.5% Triton-X was added to each well to quench the SDS. Next, 12.5 µL NEBNext High-Fidelity 2× PCR Master Mix (NEB) were added and samples were PCR-amplified using 72 °C 5 min, 98 °C 30 s, (98 °C 10 s, 63 °C 30 s, 72 °C 60 s) × 11 (bone marrow) or 12 cycles, held at 12 °C. After PCR, all wells were combined. Libraries were purified following the MinElute PCR Purification Kit manual (Qiagen) using a vacuum manifold (QIAvac 24 plus, Qiagen). Size selection was performed with SPRI Beads (Beckmann Coulter, 0.55× and 1.5×) followed by another round of SPRI Bead clean-up (Beckmann Coulter, 1.5×). Libraries were quantified using a Qubit fluorimeter (Life technologies) and the nucleosomal pattern was verified using a Tapestation (High Sensitivity D1000, Agilent). The libraries were sequenced on a HiSeq4000, NextSeq500 or NovaSeq6000 sequencer (Illumina) using custom sequencing primers with following read lengths: 50 + 10 + 12 + 50 (Read1 + Index1 + Index2 + Read2).

### snATAC-seq data alignment

Paired-end sequencing reads were demultiplexed allowing up to two mismatched to all possible barcode combinations. Reads were aligned to mm10 reference genome using bowtie2^78^ with default parameters and cell barcodes were added as a BX tag in the bam file. Only primary alignments were kept. Then we removed duplicated read pairs with Picard.^79^ Only proper read pairs with insert size less than 2000 were kept for further analysis.

### TSS enrichment calculation

Enrichment of ATAC-seq accessibility at Transcription Start Sites (TSSs) was used to assess data quality. The method for calculating enrichment at TSS was previously described here.^80^ Briefly, Tn5 corrected insertions (reads aligned to the positive strand were shifted +4 bp and reads aligned to the negative strand were shifted –5 bp) were aggregated ±2000 bp relative (TSS strand-corrected) to each unique TSS genome-wide. TSS positions were obtained from the GENCODE database vM16. Then this profile was normalized to the mean accessibility ±1900–2000 bp from the TSS and smoothed every 11 bp. The max of the smoothed profile was taken as the TSS enrichment.

### Clustering and cell type annotation

We used snapATAC package^43^ to perform read counting and cell clustering for both all-tissue clustering and tissue-level clustering. First, we removed nuclei with less than 500 fragments or TSS enrichment < 10 for all tissues (except for heart and leg muscle we used TSS enrichment cut-off of 7 to keep more usable cells). Second, we calculated a cell-by-bin matrix at 5000-bp resolution for every sample independently, binarized the matrices and subsequently merged them for each clustering task. Third, we filtered out any bins overlapping with ENCODE blacklist (mm10, http://mitra.stanford.edu/kundaje/akundaje/release/blacklists/mm10-mouse/mm10.blacklist.bed.gz). Fourth, we normalized the read coverage of all bins with log10 (count +1) and Z-score transformation, and only removed bins with absolute Z-scores higher than 2.

After these filtering steps, we calculated Jaccard Index and performed dimensional reduction using the runDiffusionMaps function on similarity matrices. The memory usage of the matrices scales quadratically with the number of nuclei. Therefore, we sampled a subset of 40,000 “landmark” nuclei to compute the matrices and then extended to the rest of the cells when the total number of nuclei exceeded 40,000 (this occurs in the clustering of all tissues, FC, LM and BM). After dimensional reduction, we selected top 20 eigenvectors based on the variance explained by each eigenvector. And then we computed 20 nearest neighbors for each nucleus and applied Leiden algorithm^81^ to define clusters. Cell clusters were annotated with 1–3 marker genes from previous publications.^82–85^ Unknown clusters dominated by low-quality cells (with low TSS enrichment scores) or doublet cells (with two marker genes and high read counts) were identified and removed.

### Detection of age-differential cCREs

For each tissue, cCREs (or ATAC-seq peaks) were called using MACS2^44^ with default parameters. Peaks overlapping with high-signal repetitive regions (specifically, _CCCTAA_n,_TTAGGG_n,GSAT-MM,SYNREP_MM from Repeatmasker annotation) were discarded. Sequencing reads from the cells of the same cell type, age and biological replicate were merged into pseudo-bulk BAM files. Then reads were counted by featureCounts function^86^ on the cCREs in the corresponding tissue. Age-differential cCREs of each cell type were identified by edgeR^46^ between 18-month and 3-month datasets with the tagwise dispersion estimator and likelihood ratio test with a *P-*value cutoff of 0.01. Different *P-*value cut-off or Benjamini–Hochberg (BH)^87^ adjusted *P-*value cutoff were also explored and did not change the main conclusions. cCREs with significant *P-*value and are more accessible in 18-month sample were denoted as age-up cCREs, while cCREs with significant *P-*value and are less accessible in 18-month sample were denoted as age-down cCREs.

As a comparison, differential cCRE analysis was also performed with edgeR on all 3 age groups, and MAST^47^ (1.20.0) using the top 20,000 regions found as differential in edgeR 3 mo vs 18 mo and 10,000 randomly sampled regions. It was impossible to run MAST on all peaks due to time and memory constraints. Age was used as a numerical variable in the linear model generated by MAST with default parameters. The comparison of all three methods is summarized in Supplementary information, Fig. S6.

To ensure fair comparison of the number of age-dependent cCREs for each cell type, we down-sampled each sample from each cell type to 1 million reads. Samples with less than 1 million reads were removed. A total of 32 cell types passed the 1 million reads threshold. Then we performed the differential cCRE analysis as stated above for each cell type. The results are summarized in Supplementary information, Fig. S8.

### Motif and gene ontology enrichment analysis

Motif enrichment analysis was performed using HOMER^88^ for the age-differential cCREs in each cell type, with non-differential cCREs as the background. Enriched gene ontology biological pathways were performed by DAVID^89^ for age-differential cCREs for each cell type as well.

### snRNA-seq experiments

Droplet-based Chromium Single Cell 3′ solution^90^ (10× Genomics, v3 (hippocampus) and v3.1 chemistry (frontal cortex)) was used to generate snRNA-seq libraries as described.^74,76^ Isolated nuclei were pelleted at 500 rcf for 5 min (4 °C; R5920, Eppendorf; acceleration/deceleration 3/3). Supernatant was removed and pellet was resuspended in 400 µL of sorting buffer: 1 mM EDTA 0.2 U/µL RNase inhibitor (Promega, N211B), 2% BSA (Sigma-Aldrich, SRE0036) in PBS and stained with DRAQ7 (1:100; Cell Signaling, 7406). 75,000 nuclei were sorted using a SH800 sorter (Sony) into 50 µL of collection buffer consisting of 1 U/µL RNase inhibitor in 5% BSA. Sorted nuclei were pelleted at 1000 rcf for 15 min (4 °C; R5920, Eppendorf; acceleration/deceleration 3/3). Nuclei were resuspended in 35 µL of reaction buffer: 0.2 U/µL RNase inhibitor (Promega, N211B), 2% BSA (Sigma-Aldrich, SRE0036) in PBS and counted on a hemocytometer. 12,000 (hippocampus) or 18,000 nuclei (frontal cortex) were loaded onto a Chromium controller (10× Genomics). Libraries were generated using the Chromium Single Cell 3′ Library Construction Kit v3 (10× Genomics; hippocampus (v3.1): PN-1000268, PN-1000120, PN-1000215; frontal cortex (v3): PN-1000075, PN-1000073, PN-120262) according to manufacturer specifications. cDNA was amplified for 12 PCR cycles. SPRISelect reagent (Beckman Coulter) was sued for size selection and clean-up steps. Library quality control was performed using Qubit dsDNA HS Assay Kit (Thermo-Fischer Scientific) and Tapestation High Sensitivity D1000 (Agilent). Libraries were sequenced using NextSeq500, HiSeq4000 or NovaSeq6000 (Illumina) with these read lengths: 28 + 8 + 91 (Read1 + Index1 + Read2).

### snRNA data processing

Cellranger^90^ version 3.0.2 was used to pre-process fastq files from 8 samples (2 replicates for each of 3-month, 10-month, and 18-month). Seurat 3.1.5^53^ was used for subsequent analysis. DoubletFinder 2.0.2^91^ was used to identify and remove doublets from each sample. Seurat’s anchor-based label transfer was used to transfer cluster labels from snRNA to the DH snATAC-seq data. Almost all cells had very high prediction scores, indicating high concordance. Since snRNA-seq and snATAC-seq had different sensitivity for defining cell clusters, we grouped cell clusters in one dataset when all of them were matched to a single cell cluster in the other dataset, to obtain consistent cell type labels for both datasets. Using the transferred labels, we defined 12 matched cell types between the RNA and ATAC data: Ogc, DG, CA1, InhN, Sub_Ent, Asc, CA2/3, Mgc, Opc, Endo, Peri, SMC. These cell-type assignments were subsequently used for gene-cCRE correlation analysis. A pseudo-bulk count table was generated by summing sequencing reads from cells of the same cell type/cluster, age and biological replicate for each gene. Age-differential genes of each cell type/cluster were then identified by edgeR^46^ between 18-month and 3-month datasets using the likelihood ratio test with an adjusted *P*-value cutoff of 0.1.

### Identification of Gene-cCRE pairs

Cells from the same matched cell types and ages (both snATAC-seq and snRNA-seq) were merged into pseudo bulks, resulting in 36 data points (12 cell type, 3 age groups) for dorsal hippocampus and 48 data points (16 cell types, 3 age groups). For every gene, we computed the weighted Pearson correlation coefficient (WPCC) between the gene transcription levels and the accessibility of any cCRE within 500 kb of the gene TSSs. The number of cells in each cell type is used as the weight, to counter the effect of outlier/extreme values in less abundant cell types. For gene annotations we used GENCODE vM 23 to be consistent with the Cellranger’s annotation. BH adjusted *P*-value cutoff of 0.05 was used to determine significant gene-cCRE pairs. Gene-cCRE pairs were then used to link age-differential cCREs to age-differential genes.

### Gaussian smoothing

We used the R package smoother to perform gaussian smoothing on the number of differential peaks (*P-*value < 0.001) within each 100 kb region of the genome (smoothing window length of 20). Regions of the genome with a high concentration of differential peaks within a short distance from each other were therefore assigned higher gaussian smoothing scores.

### Overlap with histone marks and CTCF-binding sites

We used bedtools intersect -c to overlap all called peaks for each cell type cluster with each of 7 histone ChIP-seq and CTCF ChIP-seq tracks from ENCODE. Fisher’s exact test was used to calculate the enrichment of the overlap of the ChIP-seq called regions with the top 1% of age-associated changing peaks (ordered by *P*-values calculated using edgeR comparing 3-month and 18-month samples) vs all other (not age-associated) peaks. ENCODE experiment IDs used for overlap analysis are shown in Supplementary information, Table S6. For most experiments, the ENCODE narrowPeak bed files were directly used for overlap analysis with the snATAC-seq data. For LM and HT, histone mark ChIP-seq for 5 time points (E11.5–E15.5) and 7 time points (E11.5–P0) respectively were merged to call peaks. H3K9me3 data from the forebrain were re-aligned to mm10 genome using BWA^92^ without mapping quality filter (in order not to lose any reads aligning to repetitive elements), and peaks were re-called using SICER^93^ on both ChIP-seq and input libraries.

### Hi-C data processing

To understand the three-dimensional structure of the heterochromatin domains that were reduced during aging, we downloaded Hi-C data from mouse embryonic stem cells.^94^ Reads were mapped to mm10 genome as previously described^95^ (https://github.com/ren-lab/hic-pipeline), with a mapping quality filter of 0, to allow interrogation of contacts of the repetitive regions of the genome. First principal components (PC) were computed for the Hi-C matrix. Positive and negative PCs correspond to euchromatin and heterochromatin domains.^96^

### Paired-tag experiments

Paired-tag experiments are carried out as previous described^62^ with slightly modification. After nuclei isolation with nuclei isolation buffer: 0.2% IGEPAL CA-630 (Sigma #63069), 5% BSA (Sigma #A1595) and 1 mM DTT (Invitrogen #P2325) in PBS (Invitrogen #AM9624), supplemented with 1× Proteinase Inhibitor (Roche #4693132001), 0.5 U/µL SUPERaseIn (Invitrogen #AM2696), and 0.5 U/µL RNase OUT (Invitrogen #10777019), each 300,000 of nuclei were aliquot into the 12 1.5 mL low-bind tubes. Nuclei were spin-down and resuspended in 30 µL MED#1 buffer: 20 mM HEPES (Gibco #15630080), 300 mM NaCl (Invitrogen #AM9760G), 0.5 mM Spermidine (Sigma #S2626), 1× Proteinase Inhibitor, 0.5 U/µL SUPERase In, 0.5 U/µL RNase OUT, 0.01% IGEPAL-CA630, 0.01% Digitonin (Millipore #300410), 2 mM EDTA (Invitrogen #15575020)) and keep on ice. 2 µg of H3K9me3 antibody (Abcam #ab8580) were added into 12 of 200 µL tubes containing 20 µL MED#1 buffer, and pA-Tn5 protein with 12 DNA barcodes were added and incubated at room temperature with gently rotation for 1 h. The 12 tubes of antibody-pA-Tn5 mix were then mixed with each tube of nuclei, respectively and the incubation was carried out in 4 °C with gently rotation overnight. The nuclei were then spun-down and washed two times with MED#2 buffer (20 mM HEPES, 300 mM NaCl, 0.5 mM Spermidine, 1× Proteinase Inhibitor, 0.5 U/µL SUPERase In, 0.5 U/µL RNase OUT, 0.01% IGEPAL-CA630, 0.01% Digitonin) and resuspended in 50 µL MED#2 buffer. Tagmentation reaction were activated out by adding 2 µL of 250 mM MgCl_2_ (Sigma #63069), carried out in a ThermoMixer set at 37 °C, 550 rpm for 60 min and quenched by adding 16.5 µL of 40 mM EDTA. Nuclei were then spin-down and reverse transcription were carried out with Maxima H minus reverse transcriptase (Thermo #EP0751). Nuclei were then barcoded by ligation-based combinatorial barcoding with T4 DNA Ligase (NEB #M0202L), aliquoted into 2.5–3.5k nuclei sub-libraries and lysed. Library preparation were then carried out as previous described^62^ and sequenced with read cycles 100 (read1) + 8 (index1) + 8 (index2) + 100 (read2) on a NovaSeq 6000 platform.

### Paired-tag data processing

Preprocessing of Paired-tag were carried out with the scripts available from GitHub (https://github.com/cxzhu/Paired-Tag). Briefly, cellular barcodes were extracted from Read2 and assigned to each sample barcodes (12 initial tubes for tagmentation and reverse transcription) and combination of ligated barcodes. Adaptors were trimmed from Read1 and then mapped to the reference genome with bowtie2^78^ (for DNA) and STAR^97^ (for RNA, with annotation from GENCODE GRCm38.p6). Before generating cell-counts matrices, DNA alignment files were further filtered by removing high-pileup positions (cutoff = 10). Cells with less than 500 unique H3K9me3 loci and 200 unique transcripts were removed from downstream analysis. To remove potential doublets, cells were first clustered with Seurat^53^ package based on scRNA-seq profiles with resolution = 5, cell groups with both number of DNA and RNA reads per nuclei higher than 5-fold of average reads per nuclei were excluded from further analysis. The remaining cells were again clustered with Seurat package based on scRNA-seq profiles with resolution = 0.5 and annotated based on expression level of marker genes.^62^

H3K9me3 associated domains (peaks) were called using SICER^93^ on aggregated H3K9me3 signals from Paired-tag (without input). All default parameters were used, except that window size parameter was set to 5000 and gap size was set to 10000 to detect large peaks. Peaks larger than 100Kb were kept for further downstream analysis (for instance, Fig. 6d).

### Quantifying transposable elements (TEs) expression

scTE^98^ version 1.0 was used to build a genome index for the alignment of reads to genes (gencode vM21) and TEs (rmsk mm10) using scTE_build. The scTE command was used to map reads from the unfiltered BAM files generated by Cellranger to genes and transposable element families, generating a cell by feature read count matrix. Cells with fewer than 100 genes expressed were excluded using “min_genes 100”. A pseudo-bulk count table for both genes and TEs was generated by summing reads from cells of the same cell type, age and biological replicate for each feature. Age-differential genes and TEs for each cell type were then identified by edgeR^46^ between 18-month and 3-month datasets using the likelihood ratio test. The same strategy was applied on snATAC-seq and Paired-Tag data to quantify the chromatin accessibility and H3K9me3 signal on TEs.

### Immunofluorescence staining

Eight mice were used for immunostaining experiments; the 3-month-old and 18-month-old groups included 2 male and 2 female mice each. Mice were perfused intracardially with 4% paraformaldehyde in PBS. After an overnight post-fixation in the same fixative at 4 °C, brain tissues were cut into 50 μm sections with a microtome. Brain sections were blocked with 0.3% Triton X-100 and donkey serum in PBS for 1 h at room temperature and then incubated with H3K9me3 (1:500, Abcam, ab 8898) or Lamin B1 (1:500, Abcam, Cat#229025) or L1-ORF-1p (1:200, Abcam, ab 216324) and CaMKIIα (1:300, ThermoFisher Scientific, MA1–048) primary antibody overnight at 4 °C. Next, brain sections were incubated with Alexa Fluor546-conjugated goat anti-rabbit (Invitrogen, 1:500, A-11035) or Alexa Fluor488-conjugated goat anti-mouse secondary antibodies (Invitrogen, 1:500, A-11029) for 1 h at room temperature, washed in PBS, and mounted in Vectashield containing DAPI (Vector Labs Cat#H-1500).

### Image data acquisition and quantitative fluorescence intensity analysis

After immunostaining, the sections were examined, and low- and high-power images were acquired by using a confocal microscope (FV3000, Olympus Microscopy, Japan). The slides were imaged with a 10× or 60× objective with identical settings for all matched images. Image maximum projections, *z*-stacking of sections, and cell fluorescence intensity measurements were performed by using the Fiji-ImageJ software analysis tools. We measured ~200 excitatory cells and ~100 other cells from young and aged frontal brain sections, respectively, for H3K9me3 staining. We measured ~150 excitatory cells and ~100 other cells from young and aged frontal brain sections, respectively, for Lamin B1 staining. We measured ~200 excitatory cells and ~150 other cells from young and aged frontal brain sections, respectively, for L1-ORF-1p staining. The corrected total cell fluorescence (CTCF) in an arbitrary unit (a.u.) was used for data reporting and statistical analysis.

The Linear Mixed-Effect Model (LME) has been widely used to analyze correlated data. The main idea of LME (“fitlme” in MATLAB) is to take the inherent correlations in correlated data, such as the neurons from the same mouse, into consideration when conducting statistical modeling and hypothesis testing.^99^ The LME test includes paired *t-*test and repeated-measures ANOVA as two special cases. The importance of LME and its more generalized versions has been increasingly recognized in recent studies involving large cell sample data collected from a relatively small number of animals. In this study, we used LME for data analysis shown in Fig. 7, in which measurements of staining intensity are presented based on hundreds of cells from 8 mice. We fitted an LME by using age for a fixed effect and mouse group for a random effect.

### General data processing and plots

Most of the described data-processing steps (statistical tests, clustering, plotting, and so on) were performed in Python 3.4.5 (www.python.org) and the statistical computing environment R 3.4.3 (www.r-project.org). Box plots were made with ggplot2 (https://cran.r-project.org/web/packages/ggplot2). The elements of the box plots are: center line, median; box limits, upper and lower quartiles; whiskers, 1.5× the interquartile range; points, outliers.

## Supplementary information

Supplementary Figure S1 with legend

Supplementary Figure S2 with legend

Supplementary Figure S3 with legend

Supplementary Figure S4 with legend

Supplementary Figure S5 with legend

Supplementary Figure S6 with legend

Supplementary Figure S7 with legend

Supplementary Figure S8 with legend

Supplementary Figure S9 with legend

Supplementary Figure S10 with legend

Supplementary Figure S11 with legend

Supplementary Figure S12 with legend

Supplementary Figure S13 with legend

Supplementary Figure S14 with legend

Supplementary Figure S15 with legend

Supplementary Figure S16 with legend

Supplementary Figure S17 with legend

Supplementary Table S1

Supplementary Table S2

Supplementary Table S3

Supplementary Table S4

Supplementary Table S5

Supplementary Table S6

## Data Availability

All sequencing datasets have been deposited in the Gene Expression Omnibus repository with the accession number GSE187332.

## Code availability

Scripts are available at https://github.com/shawnzhangyx/mouse_aging.
