# Supplementary material for: Single-cell epigenome analysis reveals age-associated decay of heterochromatin domains in excitatory neurons in the mouse brain
Source: Cell Res. 2022 Oct 7;32(11):1008–21. doi: 10.1038/s41422-022-00719-6 (PMC9652396; doi:10.1038/s41422-022-00719-6)
Supplement: Supplementary file 1 — Supplementary Figure S1 with legend [file 41422_2022_719_MOESM1_ESM.pdf]

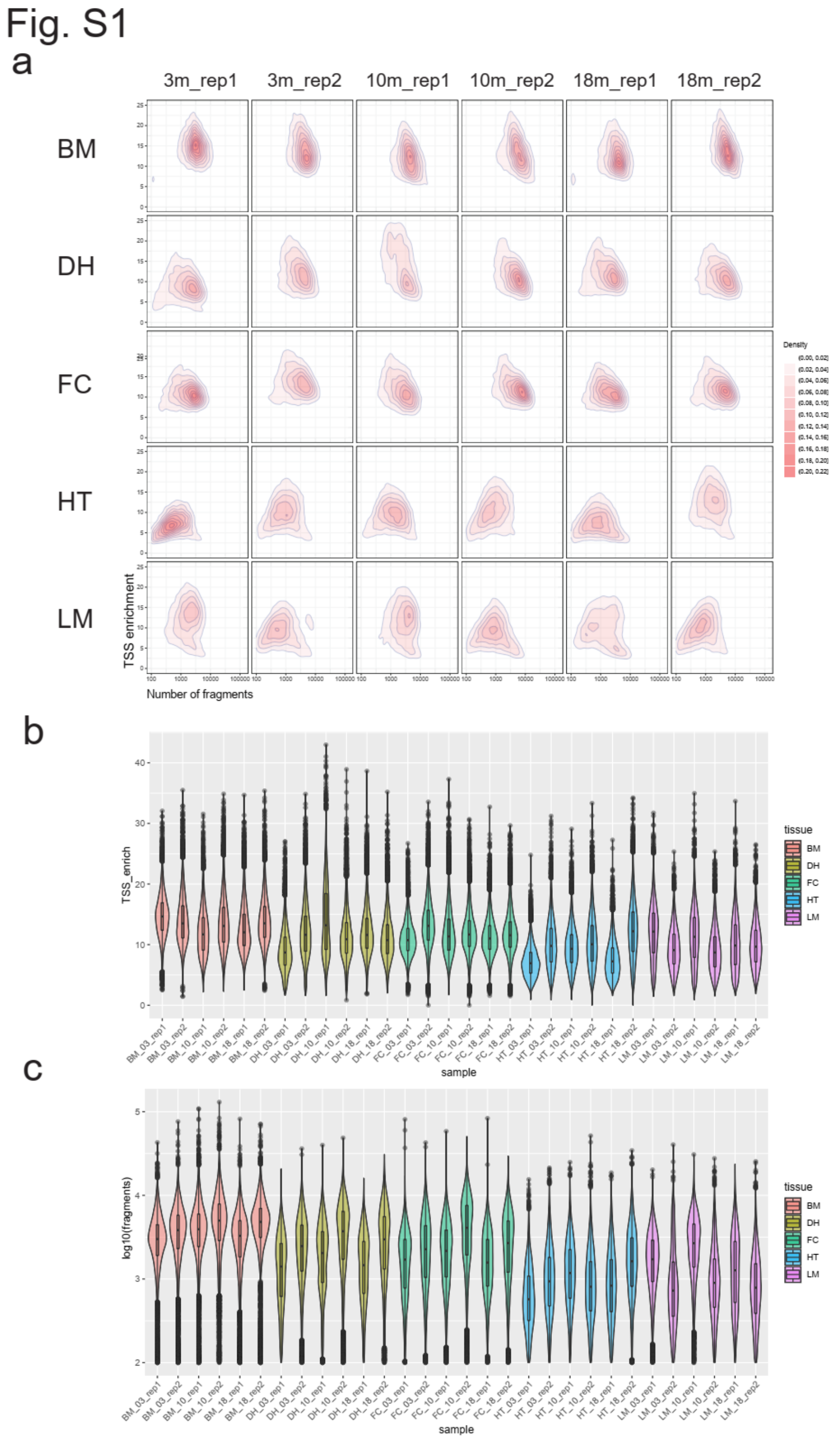

Figure. S1. Quality control metrics of snATAC-seq data collected in the present study. a) Density plots showing the distributions of number of fragments and TSS enrichment scores for all samples. **b)** Violin plots and boxplots showing the distribution of TSS enrichment scores for all samples. **c)** Violin plots and boxplots showing the distribution of log10 (Number of fragments) from all samples.
