# Supplementary material for: Single-cell epigenome analysis reveals age-associated decay of heterochromatin domains in excitatory neurons in the mouse brain
Source: Cell Res. 2022 Oct 7;32(11):1008–21. doi: 10.1038/s41422-022-00719-6 (PMC9652396; doi:10.1038/s41422-022-00719-6)
Supplement: Supplementary file 2 — Supplementary Figure S2 with legend [file 41422_2022_719_MOESM2_ESM.pdf]

Fig.S2

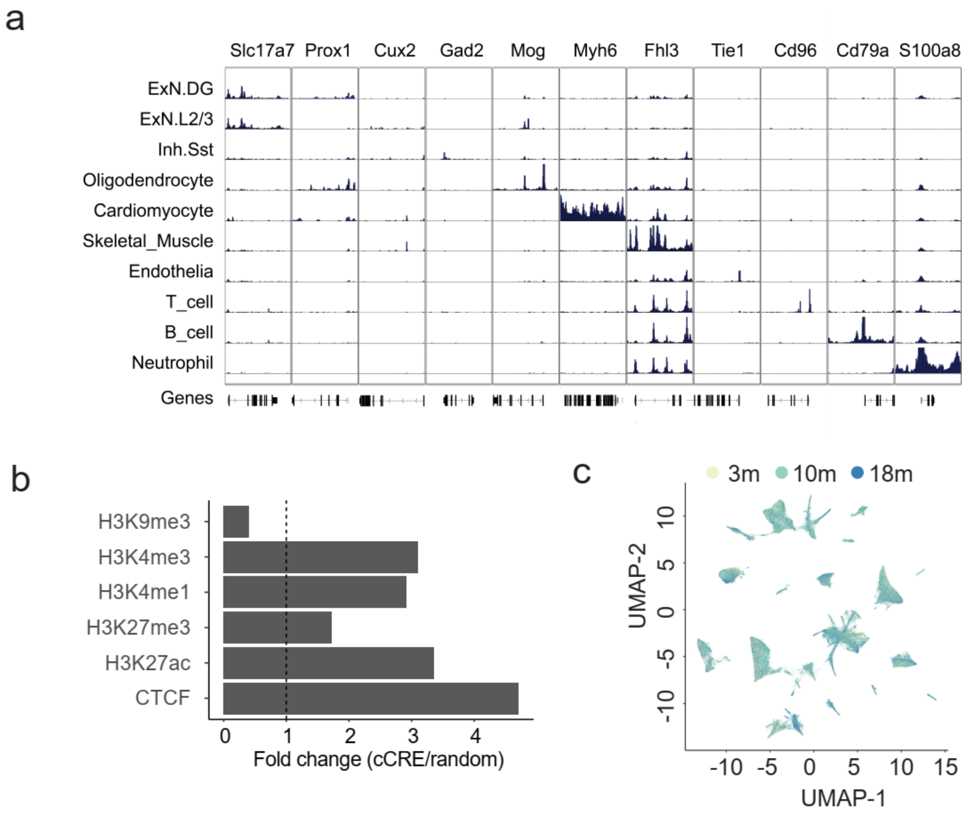

Figure. S2. Annotation of cell types and identification of cCREs from snATAC-seq data. a) Genome browser view showing the chromatin accessibility signal at the promoters of different genes for a few representative cell types. **b)** Barplot showing the fold enrichment of histone marks or CTCF on cCREs over random genomic segments of the same size. **c)** UMAP plot showing the distribution of cells by age.
