# Supplementary material for: Single-cell epigenome analysis reveals age-associated decay of heterochromatin domains in excitatory neurons in the mouse brain
Source: Cell Res. 2022 Oct 7;32(11):1008–21. doi: 10.1038/s41422-022-00719-6 (PMC9652396; doi:10.1038/s41422-022-00719-6)
Supplement: Supplementary file 3 — Supplementary Figure S3 with legend [file 41422_2022_719_MOESM3_ESM.pdf]

Fig. S3

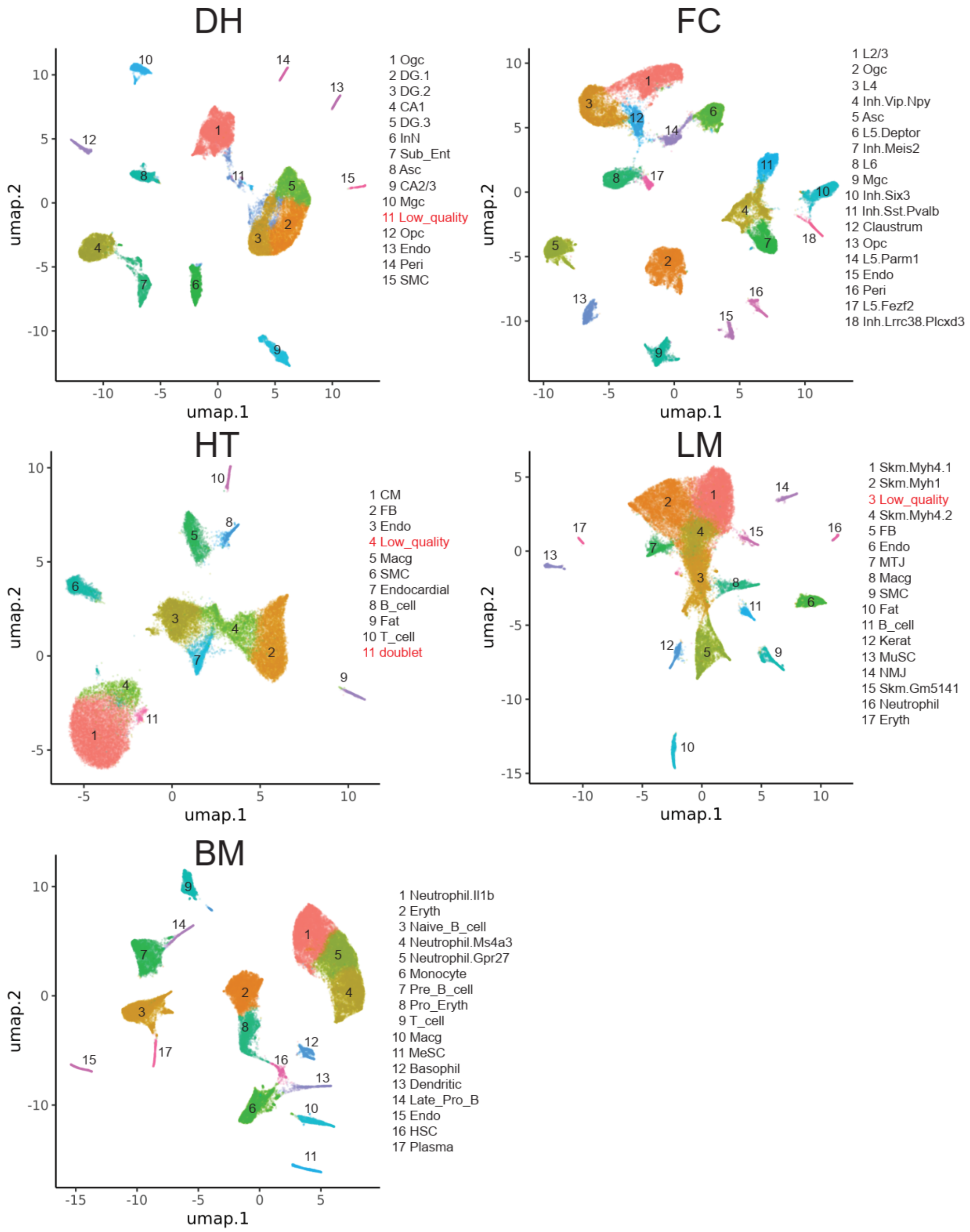

Figure. S3. Tissue-level clustering results. UMAP dimensional reduction plots showing the cell clustering and cell type annotations for all tissues.
