# Supplementary material for: Single-cell epigenome analysis reveals age-associated decay of heterochromatin domains in excitatory neurons in the mouse brain
Source: Cell Res. 2022 Oct 7;32(11):1008–21. doi: 10.1038/s41422-022-00719-6 (PMC9652396; doi:10.1038/s41422-022-00719-6)
Supplement: Supplementary file 4 — Supplementary Figure S4 with legend [file 41422_2022_719_MOESM4_ESM.pdf]

Fig.S4

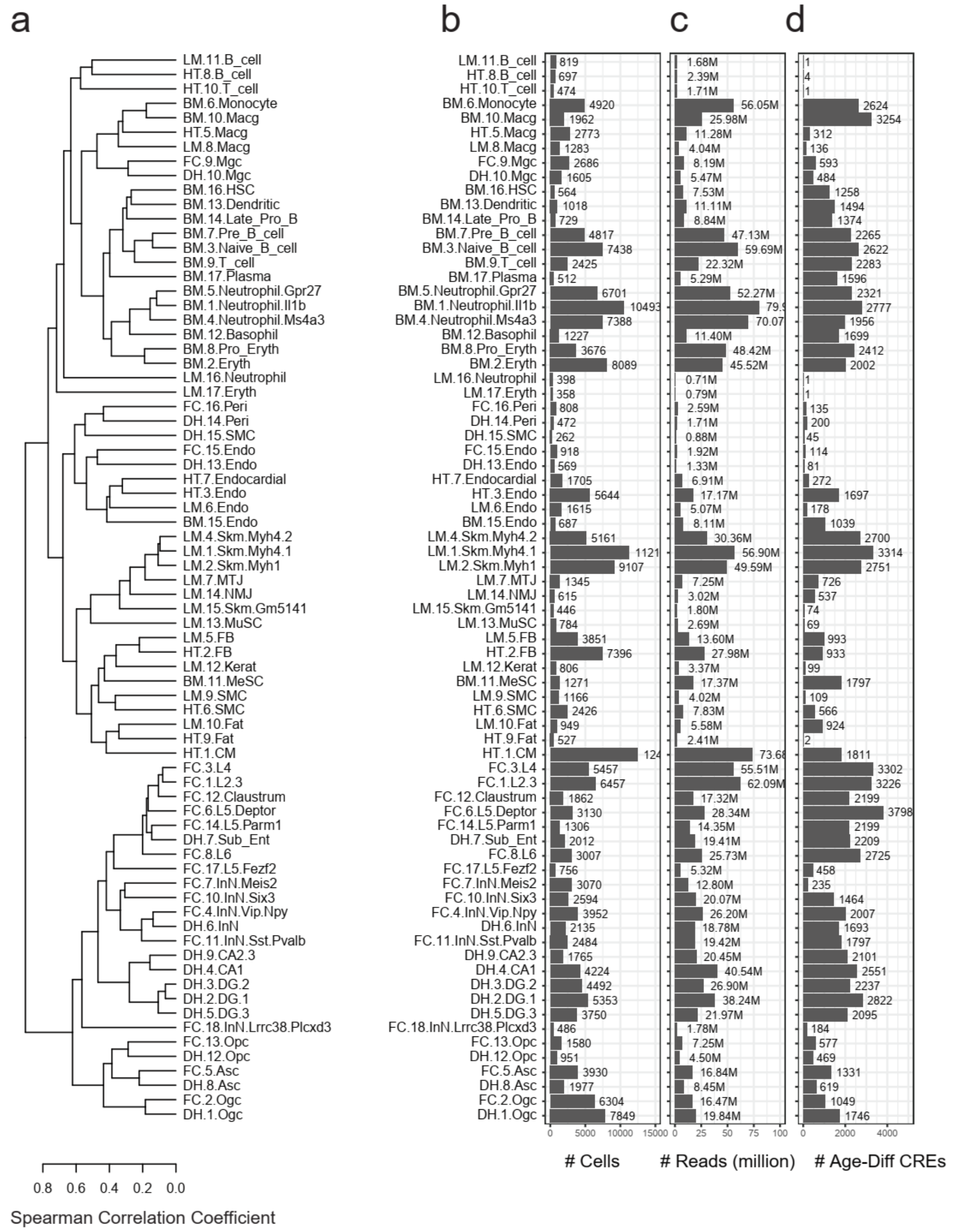

Figure. S4. Hierarchical clustering results and metrics (sequencing depth and number of age-dependent cCREs) for all cell types. a) Hierarchical clustering of all cell types based on 1- Spearman Correlation Coefficient. **b)** Barplot showing the number of cells for each cell type. **c)** Barplot showing the number of reads for each cell type. **d)** Barplot showing the number of age-differential cCREs detected for each cell type.
