# Supplementary material for: Single-cell epigenome analysis reveals age-associated decay of heterochromatin domains in excitatory neurons in the mouse brain
Source: Cell Res. 2022 Oct 7;32(11):1008–21. doi: 10.1038/s41422-022-00719-6 (PMC9652396; doi:10.1038/s41422-022-00719-6)
Supplement: Supplementary file 5 — Supplementary Figure S5 with legend [file 41422_2022_719_MOESM5_ESM.pdf]

Fig.S5

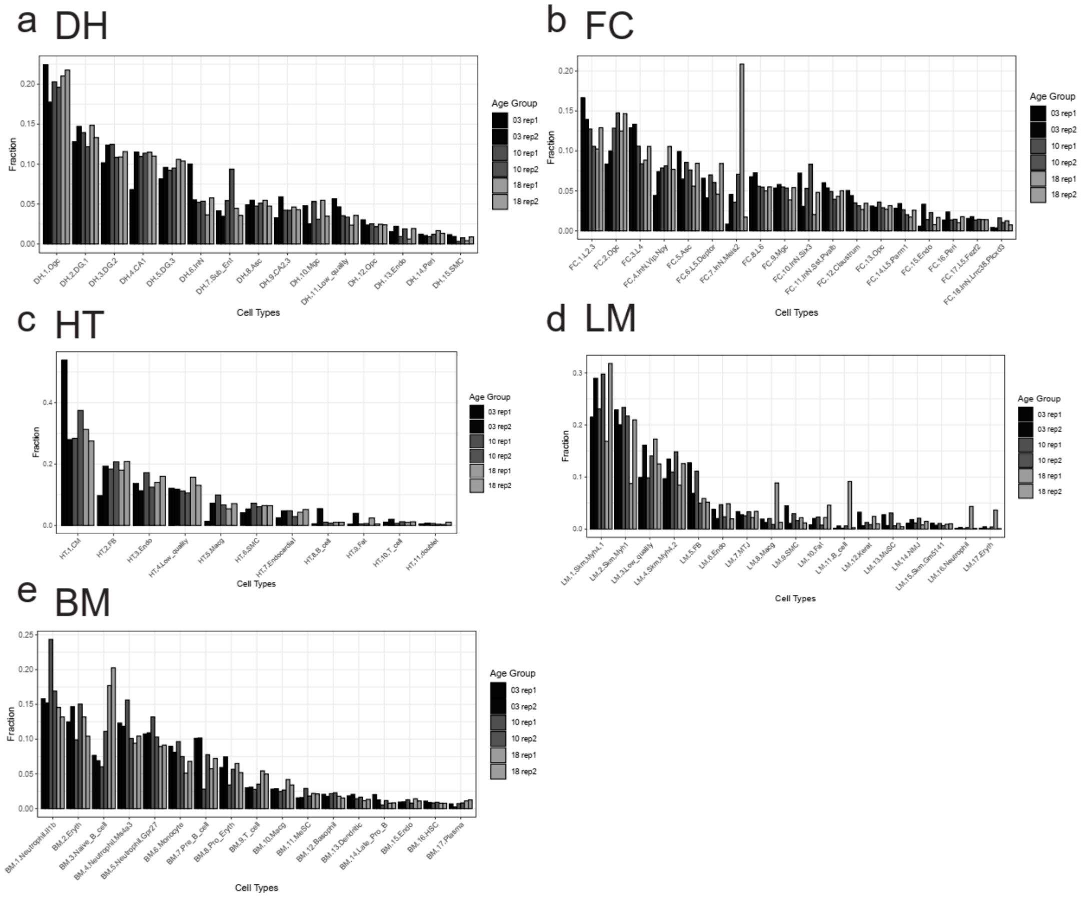

Figure. S5. Changes in the fraction of cell types in all five tissues. (a-e) Barplot showing the cell-type compositional changes in each tissue.
