# Supplementary material for: Single-cell epigenome analysis reveals age-associated decay of heterochromatin domains in excitatory neurons in the mouse brain
Source: Cell Res. 2022 Oct 7;32(11):1008–21. doi: 10.1038/s41422-022-00719-6 (PMC9652396; doi:10.1038/s41422-022-00719-6)
Supplement: Supplementary file 6 — Supplementary Figure S6 with legend [file 41422_2022_719_MOESM6_ESM.pdf]

Fig. S6

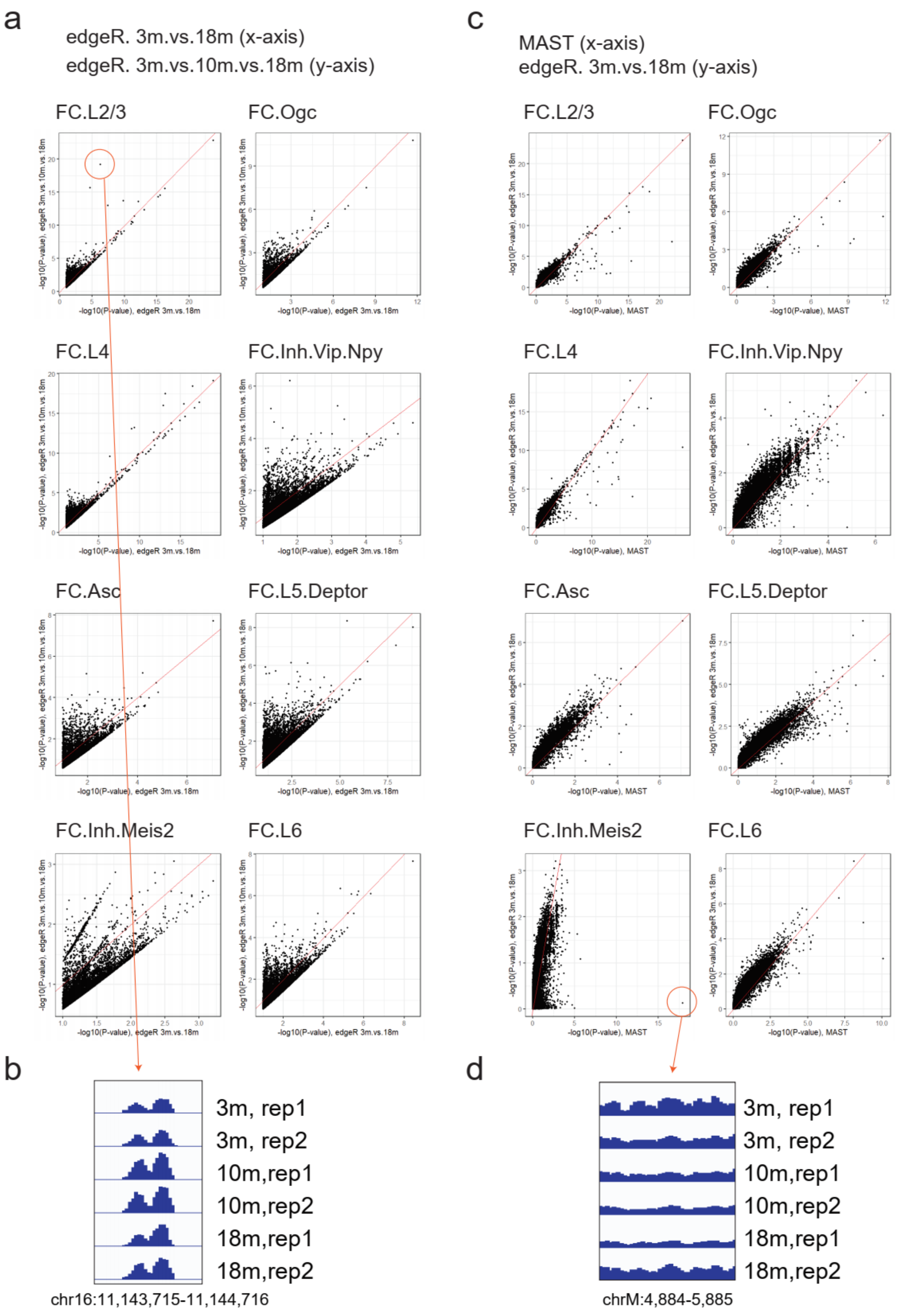
a
edgeR. 3m.vs.18m (x-axis)
edgeR. 3m.vs.10m.vs.18m (y-axis)
FC.L2/3
FC.Ogc
FC.L4
FC.Inh.Vip.Npy
FC.Asc
FC.L5.Deptor
FC.Inh.Meis2
FC.L6
-log10(P-value), edgeR 3m.vs.18m
b
3m, rep1
3m, rep2
10m,rep1
10m,rep2
18m,rep1
18m,rep2
chr16:11,143,715-11,144,716
c
MAST (x-axis)
edgeR. 3m.vs.18m (y-axis)
FC.L2/3
FC.Ogc
FC.L4
FC.Inh.Vip.Npy
FC.Asc
FC.L5.Deptor
FC.Inh.Meis2
FC.L6
-log10(P-value), MAST
d
3m, rep1
3m, rep2
10m,rep1
10m,rep2
18m,rep1
18m,rep2
chrM:4,884-5,885

Figure. S6. Comparison of different computational approaches to detect age-dependent cCREs. a) Scatterplots showing the negative logarithmic transformed P-value from edgeR analysis comparing chromatin accessibility of each cell type from 3-month to 18-month, and comparing all three age groups. Statistical tests are performed on the top 8 most abundant cell types in frontal cortex. **b)** Genome browser view showing the ATAC-seq signals at genomic regions ranked more favorably by edgeR three age group comparisons. **c)** Scatterplots showing the - negative logarithmic transformed p-value of edgeR analysis comparing chromatin accessibility in cell types from 3-month and 18-month, and negative logarithmic transformed p-value of MAST using age as dependent variable. The tests are performed on the top 8 most abundant cell types in frontal cortex. **d)** Genome browser view showing the ATAC-seq signals at regions ranked more favorably by MAST.
