# Supplementary material for: Single-cell epigenome analysis reveals age-associated decay of heterochromatin domains in excitatory neurons in the mouse brain
Source: Cell Res. 2022 Oct 7;32(11):1008–21. doi: 10.1038/s41422-022-00719-6 (PMC9652396; doi:10.1038/s41422-022-00719-6)
Supplement: Supplementary file 7 — Supplementary Figure S7 with legend [file 41422_2022_719_MOESM7_ESM.pdf]

Fig. S7

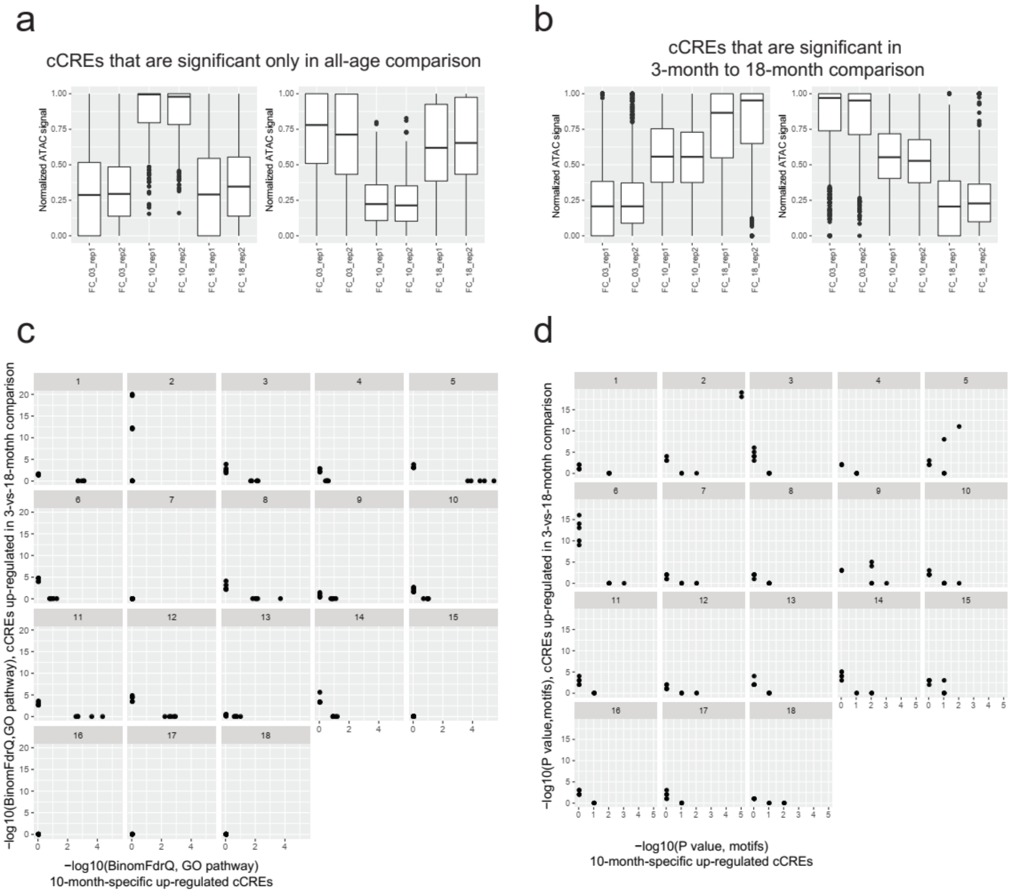

Figure. S7. cCREs that are unique to 10-month datasets. a) boxplot showing the normalized ATAC-seq signal of cCREs that are found to be only significant in edgeR all-age-group comparison but not in 3m-vs-18m comparison. Result is from layer 2/3 cortical excitatory neurons. **b)** boxplot showing the normalized ATAC-seq signal of cCREs that are significant in edgeR 3m-vs-18m comparison. Result is from layer 2/3 cortical excitatory neurons. **c)** Scatterplot showing the -log10 adjusted p-value of GO term analysis of 10-month-specific and 3m-vs-18m cCREs. Each data point is a GO term. Each subpanel is a cell type from the frontal cortex. **d)** Scatterplot showing the -log10 adjusted p-value of motif analysis of 10-month-specific and 3m-vs-18m cCREs. Each data point is a motif. Each subpanel is a cell type from the frontal cortex.
