# Supplementary material for: Single-cell epigenome analysis reveals age-associated decay of heterochromatin domains in excitatory neurons in the mouse brain
Source: Cell Res. 2022 Oct 7;32(11):1008–21. doi: 10.1038/s41422-022-00719-6 (PMC9652396; doi:10.1038/s41422-022-00719-6)
Supplement: Supplementary file 8 — Supplementary Figure S8 with legend [file 41422_2022_719_MOESM8_ESM.pdf]

Fig. S8

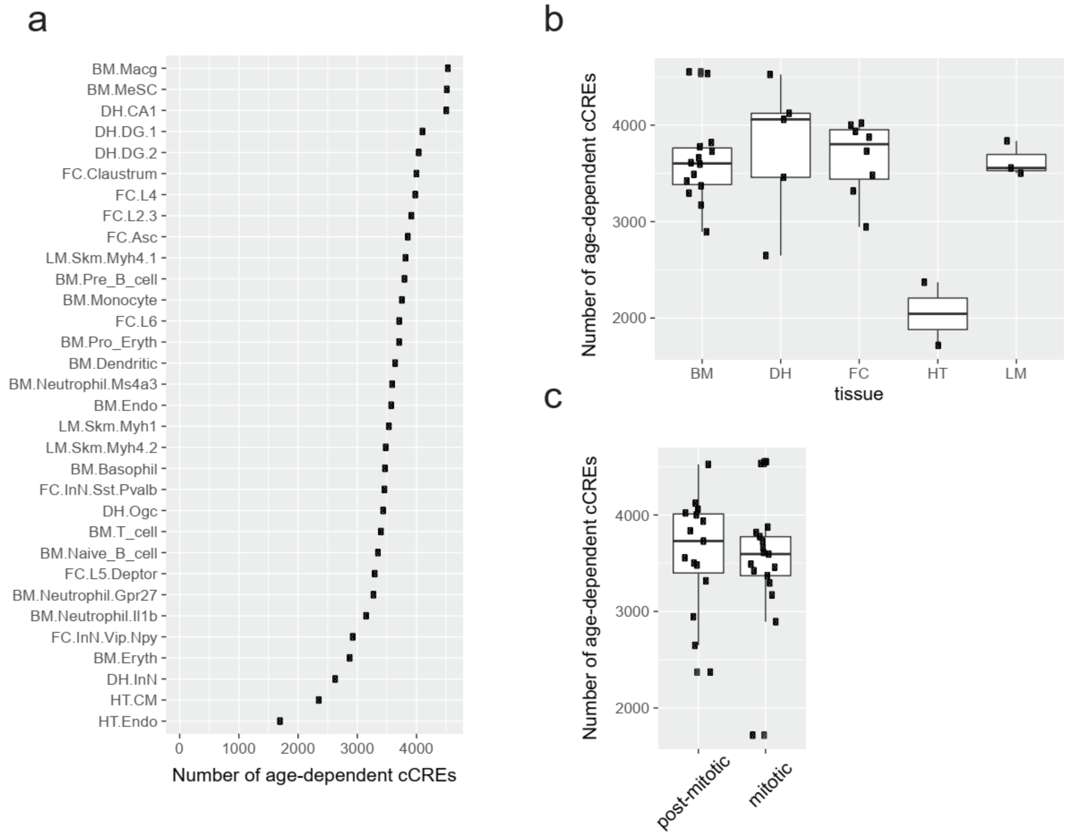

Figure. S8. Number of age-dependent cCREs in each cell type after sampling down to 1 million reads per sample. a) Scatter plot showing the number of age-dependent cCREs for each cell type. **b)** Boxplot showing the number of age-dependent cCREs for each tissue. **c)** Boxplot showing the number of age-dependent cCREs for mitotic and post-mitotic cells.
