# Supplementary material for: Single-cell epigenome analysis reveals age-associated decay of heterochromatin domains in excitatory neurons in the mouse brain
Source: Cell Res. 2022 Oct 7;32(11):1008–21. doi: 10.1038/s41422-022-00719-6 (PMC9652396; doi:10.1038/s41422-022-00719-6)
Supplement: Supplementary file 9 — Supplementary Figure S9 with legend [file 41422_2022_719_MOESM9_ESM.pdf]

Fig.S9

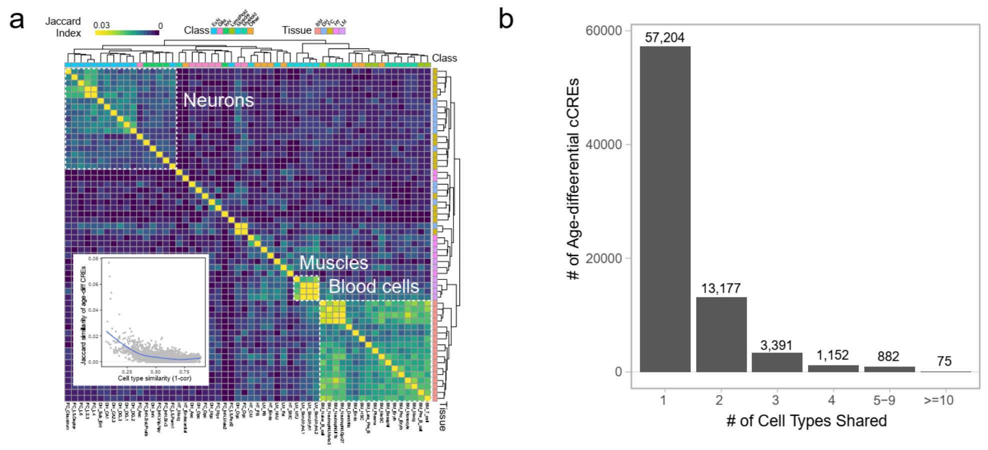

Figure. S9. Age-dependent cCREs are mostly cell-type-specific and tend to be shared among similar cell types. a) Heatmap showing the pairwise Jaccard Similarity Index of age-dependent cCREs between different cell types. On the bottom left is a scatter plot showing the pairwise Jaccard Index of age-dependent cCREs (y-axis) and pairwise 1-Spearman Correlation Coefficients of their genome-wide accessibility (x-axis) between different cell types. **b)** Barplot showing the number of age-dependent cCREs, grouped by the number of cell types in which they were found.
