# Supplementary material for: Single-cell epigenome analysis reveals age-associated decay of heterochromatin domains in excitatory neurons in the mouse brain
Source: Cell Res. 2022 Oct 7;32(11):1008–21. doi: 10.1038/s41422-022-00719-6 (PMC9652396; doi:10.1038/s41422-022-00719-6)
Supplement: Supplementary file 10 — Supplementary Figure S10 with legend [file 41422_2022_719_MOESM10_ESM.pdf]

Fig. S10

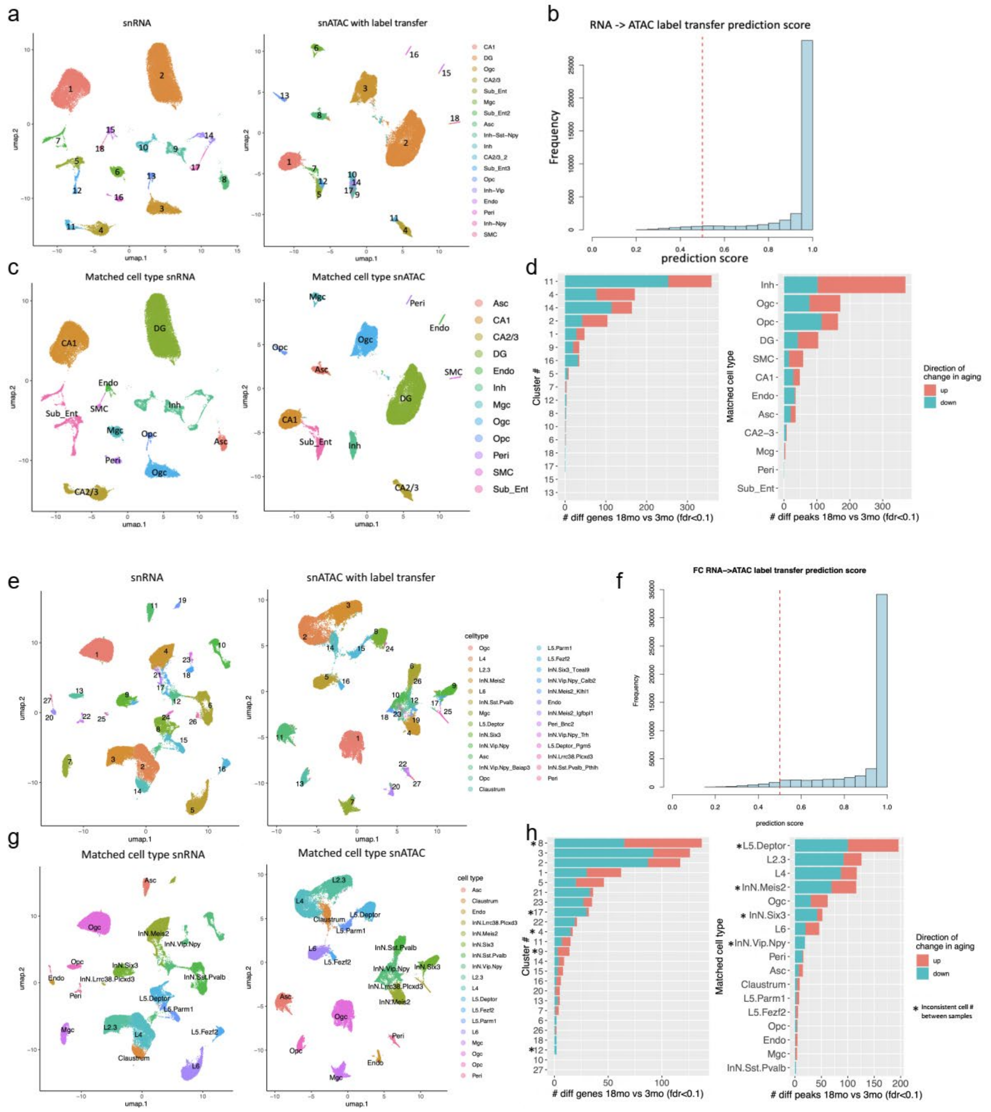

Figure. S10. Clustering and differential expression analysis of snRNA-seq datasets and their integration of snATAC-seq datasets. a) UMAP plot showing the projection of single cell in the transcriptomic space (left) and ATAC-seq space with the label transferred from snRNA-seq (right). **b)** Barplot showing the distribution of RNA-to-ATAC label transfer prediction score. **c)** UMAP plot showing the projection of single cell in the transcriptomic space (left) and ATAC-seq space with the label transferred from snRNA-seq (right) with consistent cell type labels that merge one-to-many matches. **d)** Boxplot showing the number of age-differential genes for each cell type. Left: cell types defined with snRNA-seq data; right: matched and consistent cell type labels from snRNA-seq and snATAC-seq data. **e-h)** Same panels as a-d) but for frontal cortex. **h)** *star denotes cell types with large variability in cell numbers between age groups.
