# Supplementary material for: Single-cell epigenome analysis reveals age-associated decay of heterochromatin domains in excitatory neurons in the mouse brain
Source: Cell Res. 2022 Oct 7;32(11):1008–21. doi: 10.1038/s41422-022-00719-6 (PMC9652396; doi:10.1038/s41422-022-00719-6)
Supplement: Supplementary file 11 — Supplementary Figure S11 with legend [file 41422_2022_719_MOESM11_ESM.pdf]

Fig. S11

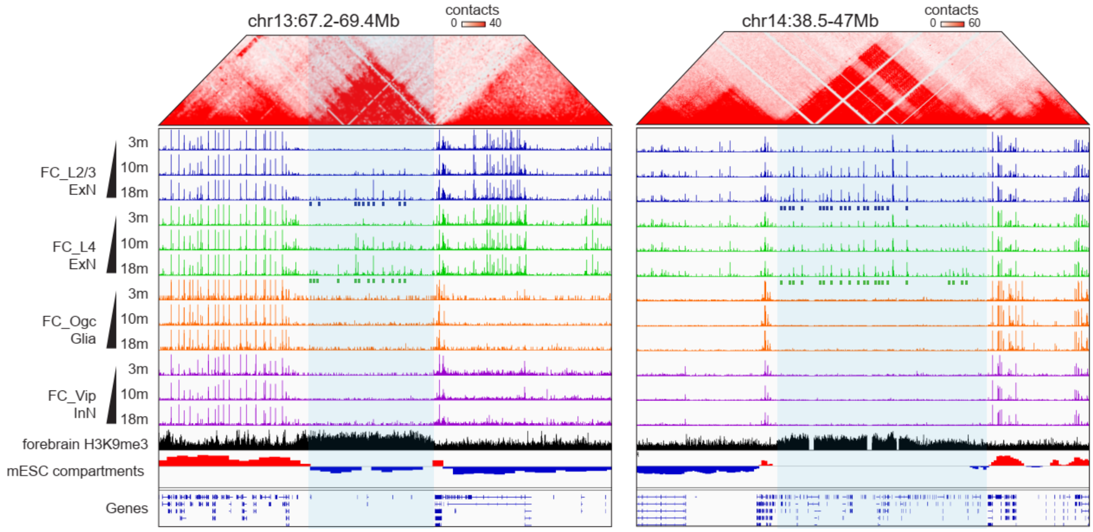

Figure. S11. Additional examples of age-up cCRE clusters that overlapped with H3K9me3 domains. Genome browser view of the ATAC-seq signals of brain cell types, H3K9me3 signal from post-natal forebrain and compartment (first principal component from Hi-C) and Hi-C matrix from mouse embryonic stem cells. Blue and green rectangles indicate locations of age-up cCREs in corresponding cell types.
