# Supplementary material for: Single-cell epigenome analysis reveals age-associated decay of heterochromatin domains in excitatory neurons in the mouse brain
Source: Cell Res. 2022 Oct 7;32(11):1008–21. doi: 10.1038/s41422-022-00719-6 (PMC9652396; doi:10.1038/s41422-022-00719-6)
Supplement: Supplementary file 12 — Supplementary Figure S12 with legend [file 41422_2022_719_MOESM12_ESM.pdf]

Fig. S12

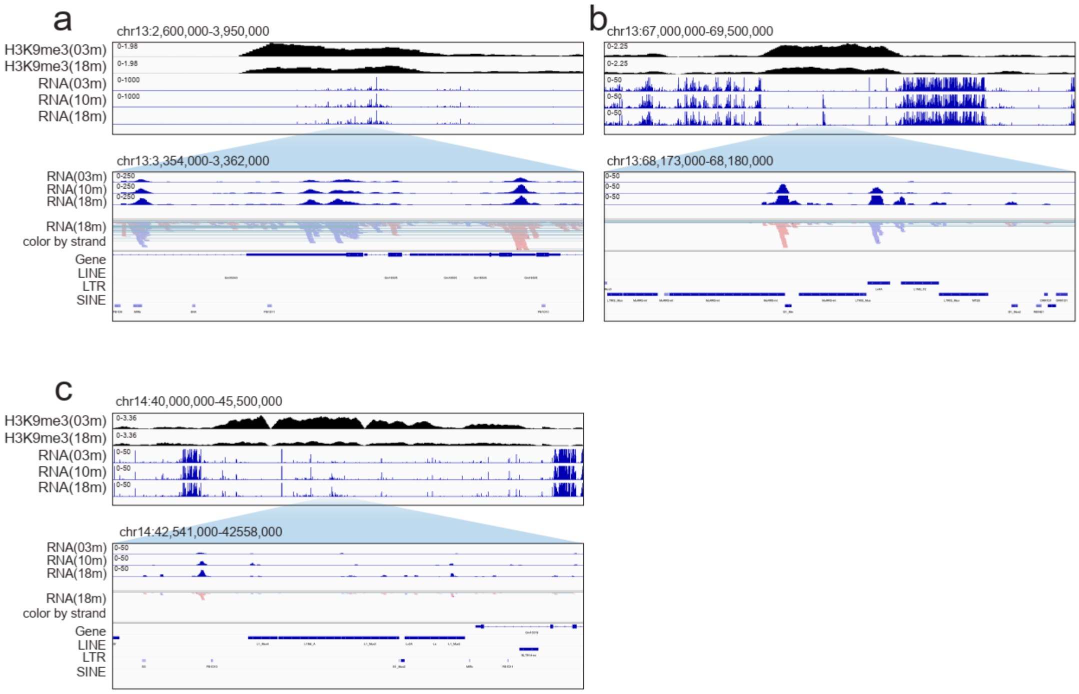

Figure. S12. Changes in transcription at H3K9me3 domains that were lost in layer 2/3 cortical neurons. a-c) Genome browser views of three representative regions showing the H3K9me3 (from Paired-tag) and RNA signals (from 10x genomics snRNA-seq) at pseudogene **(a)**, repetitive elements **(b)** and unannotated sequence **(c)**. For RNA signal track colored by strand, red color indicates forward strand and blue indicates reverse strand.
