# Supplementary material for: Single-cell epigenome analysis reveals age-associated decay of heterochromatin domains in excitatory neurons in the mouse brain
Source: Cell Res. 2022 Oct 7;32(11):1008–21. doi: 10.1038/s41422-022-00719-6 (PMC9652396; doi:10.1038/s41422-022-00719-6)
Supplement: Supplementary file 13 — Supplementary Figure S13 with legend [file 41422_2022_719_MOESM13_ESM.pdf]

Fig. S13

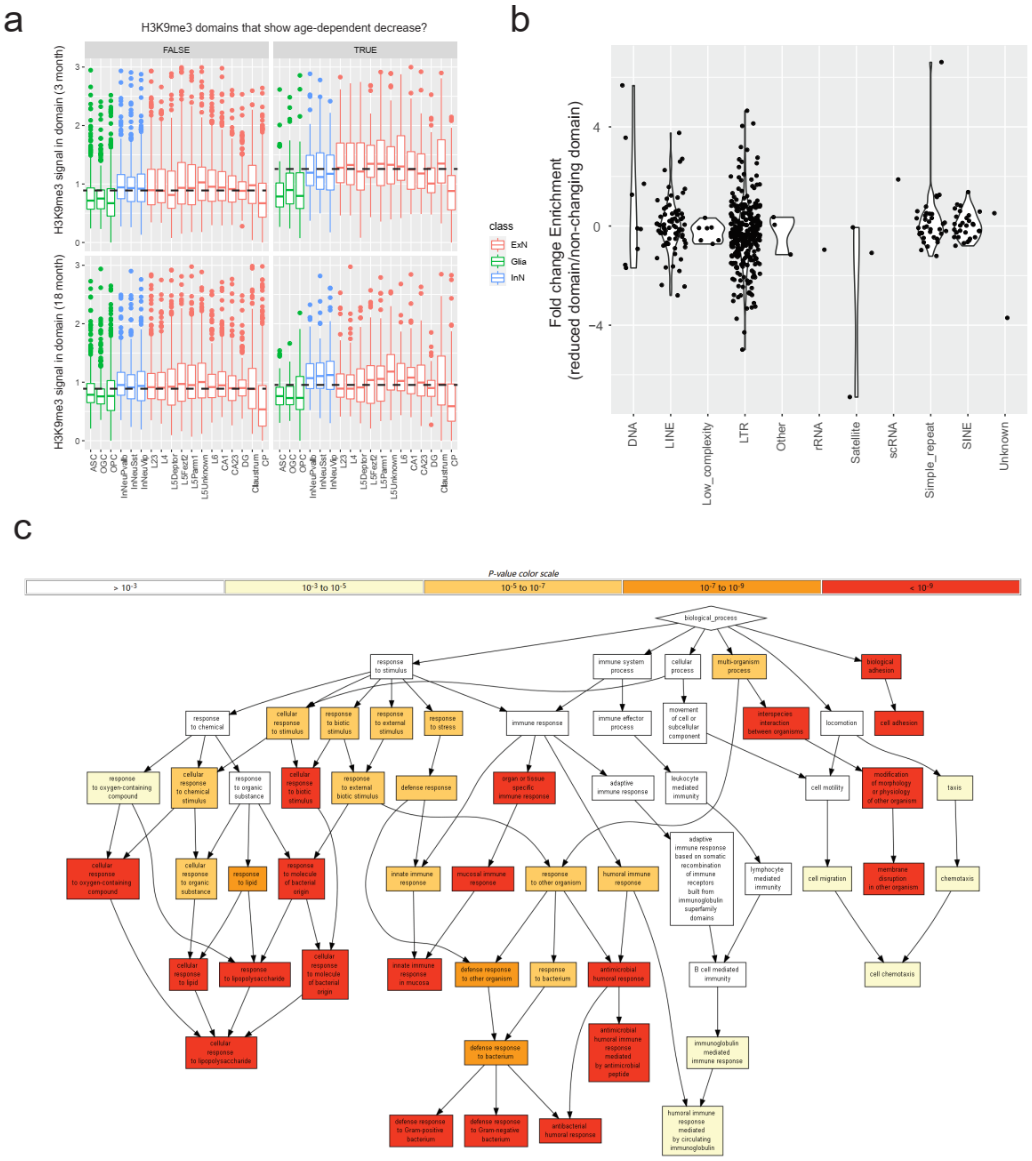

Figure. S13. Features of the heterochromatin domains that are reduced during aging. a) Boxplots showing H3K9me3 signals in heterochromatin domains in 3-month (upper panel) and 18-month (lower panel) samples. The panels on the right indicate the domains with reduced H3K9me3 during aging. The black dashed lines denote the average level of H3K9me3 for excitatory neurons in that sub panel. **b)** Violin plots showing the fold enrichment of different types of TEs in reduced heterochromatin domains. Each data point is a type of TE. **c)** GO term enrichment results comparing the genes within the reduced heterochromatin domain to that of non-changing heterochromatin domains.
