# Supplementary material for: Single-cell epigenome analysis reveals age-associated decay of heterochromatin domains in excitatory neurons in the mouse brain
Source: Cell Res. 2022 Oct 7;32(11):1008–21. doi: 10.1038/s41422-022-00719-6 (PMC9652396; doi:10.1038/s41422-022-00719-6)
Supplement: Supplementary file 14 — Supplementary Figure S14 with legend [file 41422_2022_719_MOESM14_ESM.pdf]

Fig. S14

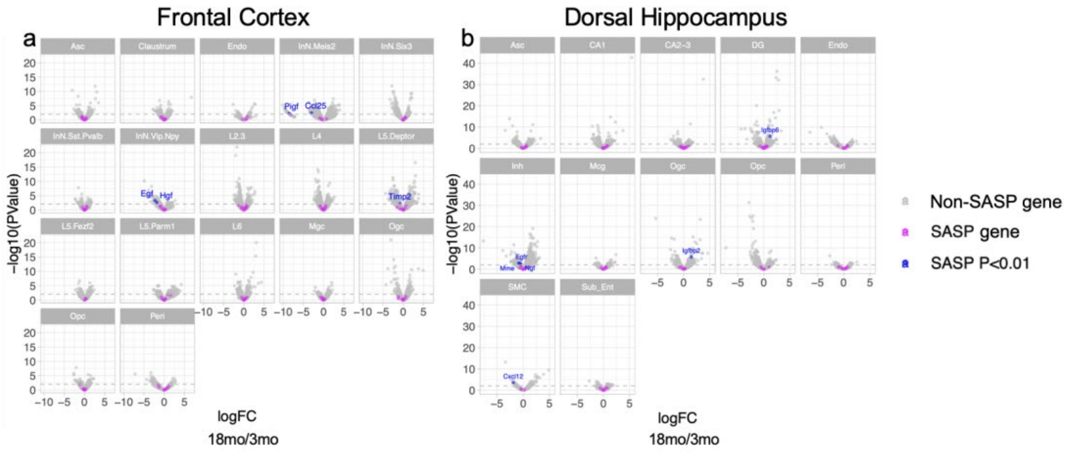

Figure. S14. Negligible change in the expression of senescence marker genes in aged excitatory neurons. a, b) Volcano plots showing the log fold change of 18-month over 3-month samples and -log10 p-value of a few senescence marker genes in frontal cortex and dorsal hippocampus.
