# Supplementary material for: Single-cell epigenome analysis reveals age-associated decay of heterochromatin domains in excitatory neurons in the mouse brain
Source: Cell Res. 2022 Oct 7;32(11):1008–21. doi: 10.1038/s41422-022-00719-6 (PMC9652396; doi:10.1038/s41422-022-00719-6)
Supplement: Supplementary file 15 — Supplementary Figure S15 with legend [file 41422_2022_719_MOESM15_ESM.pdf]

Fig. S15

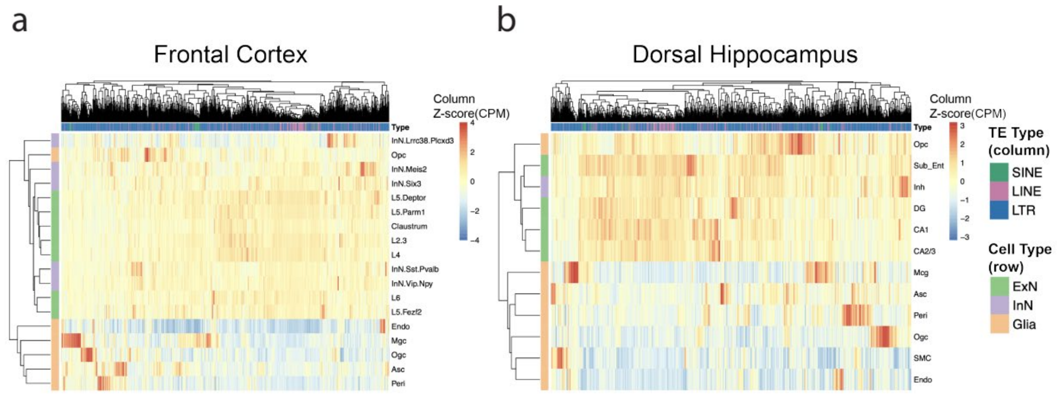

Figure. S15. Expression levels of transposable elements. a,b) Heatmaps showing the transcriptional level of transposable elements in various cell types in frontal cortex and dorsal hippocampus. The expression level is represented by count per million (CPM) and then Z-score transformed by column (one repeat subfamily).
