# Supplementary material for: Single-cell epigenome analysis reveals age-associated decay of heterochromatin domains in excitatory neurons in the mouse brain
Source: Cell Res. 2022 Oct 7;32(11):1008–21. doi: 10.1038/s41422-022-00719-6 (PMC9652396; doi:10.1038/s41422-022-00719-6)
Supplement: Supplementary file 16 — Supplementary Figure S16 with legend [file 41422_2022_719_MOESM16_ESM.pdf]

Fig.S16

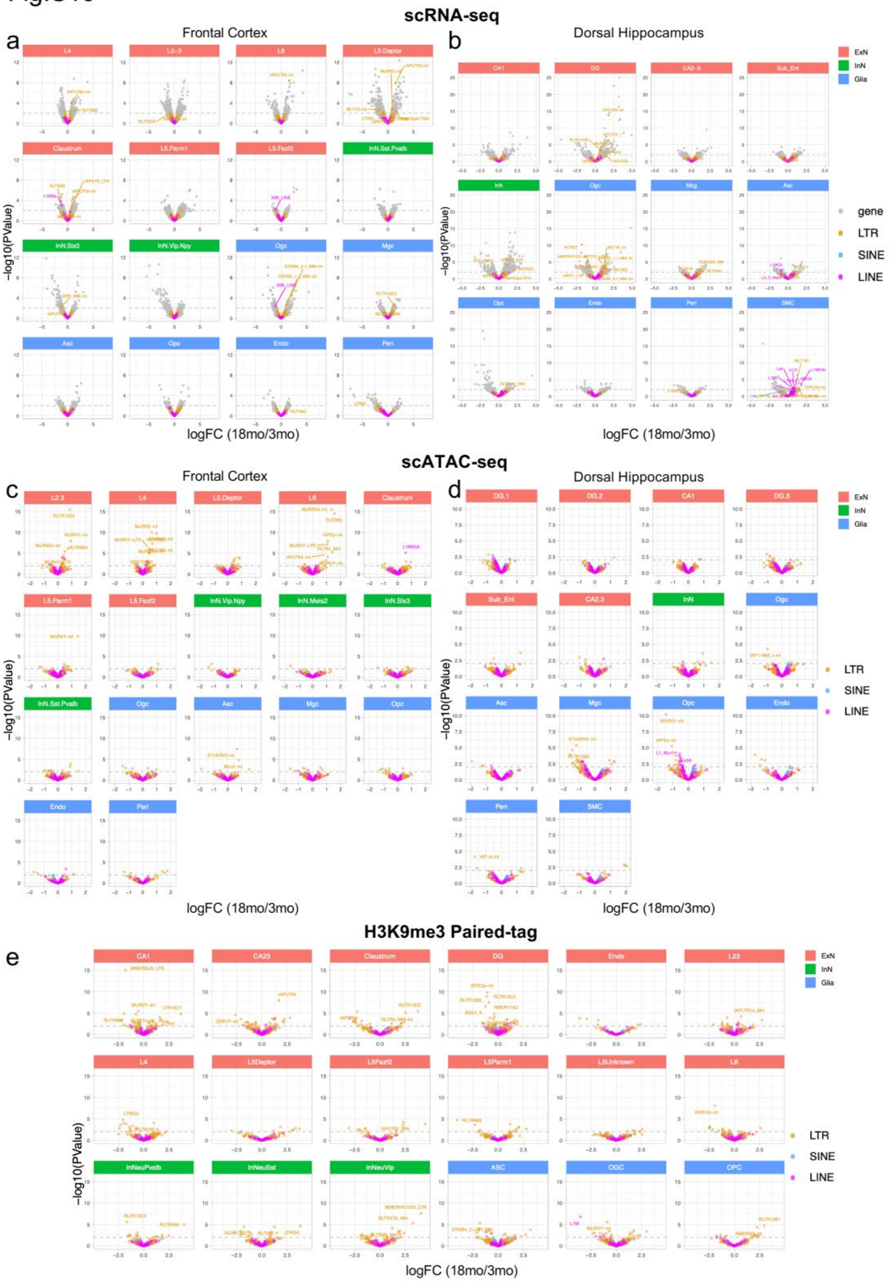

Figure. S16. Age-dependent changes in transcription, chromatin accessibility and H3K9me3 heterochromatin signal of transposable elements. a,b) Volcano plots showing the log10 p-value and log fold change of transcription (18 month over 3 month) of genes and transposable elements. Dash lines mark p-value cut-off of 0.01. **c,d)** Volcano plots showing the log10 p-value and log fold change of chromatin accessibility (18 month over 3 month) of transposable elements. Dash lines mark p-value cut-off of 0.01. **e)** Volcano plots showing the log10 p-value and log fold change of H3K9me3 signal from Paired-Tag (18 month over 3 month) of transposable elements. Dash lines mark p-value cut-off of 0.01.
