# Supplementary material for: Single-cell epigenome analysis reveals age-associated decay of heterochromatin domains in excitatory neurons in the mouse brain
Source: Cell Res. 2022 Oct 7;32(11):1008–21. doi: 10.1038/s41422-022-00719-6 (PMC9652396; doi:10.1038/s41422-022-00719-6)
Supplement: Supplementary file 17 — Supplementary Figure S17 with legend [file 41422_2022_719_MOESM17_ESM.pdf]

Fig. S17

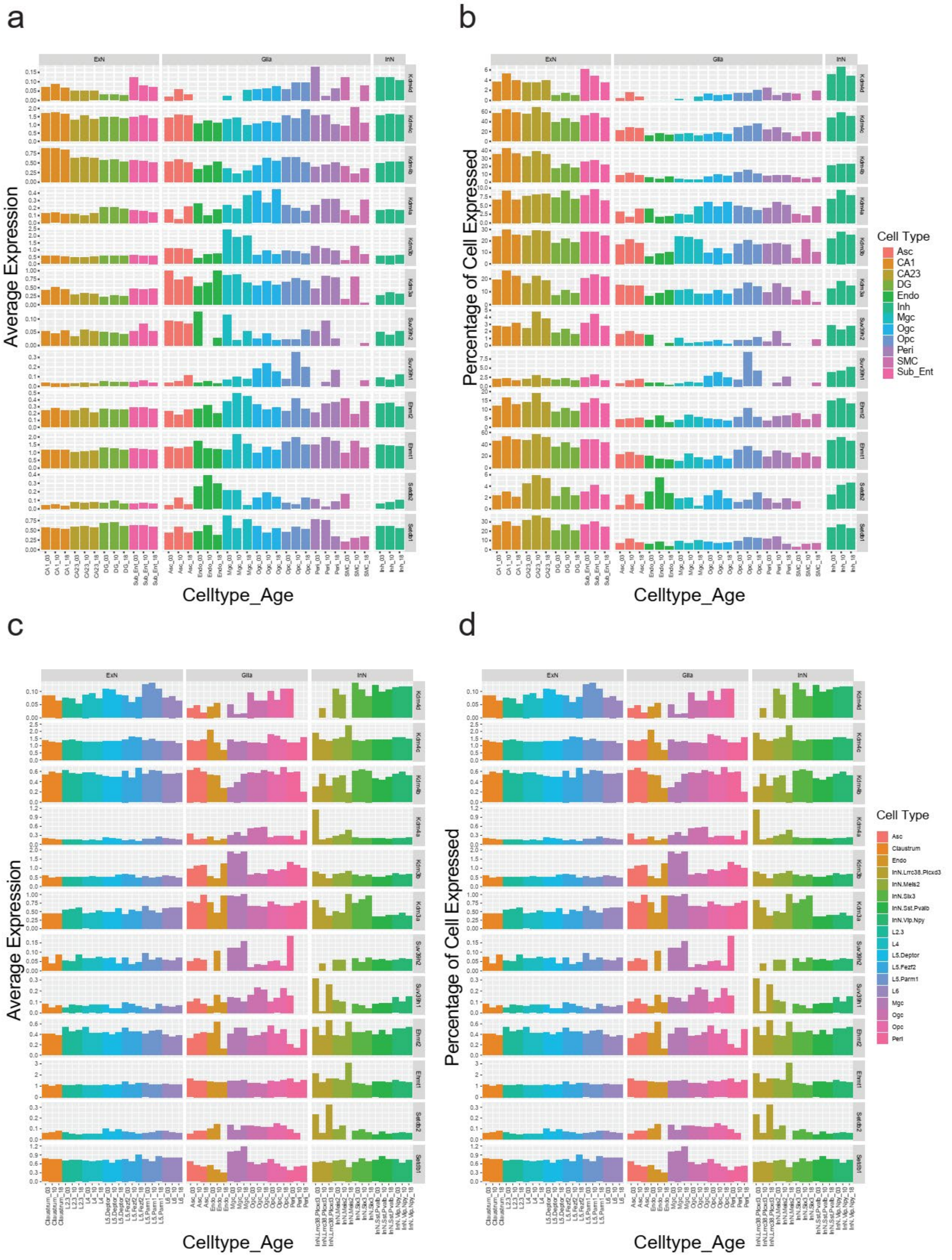

Figure. S17. Transcriptional levels of epigenetic regulators of H3K9me3 are not altered during aging. Barplots showing the average single-cell expression of various epigenetic regulators of H3K9me3 in dorsal hippocampus **(a)** and frontal cortex **(c)**, and percentage of cells expressing these regulators in dorsal hippocampus **(b)** and frontal cortex **(d)**.
